# A Rational Framework to Estimate the Chiroptical Activity of [6]Helicene Derivatives

**DOI:** 10.1021/acs.jpca.5c04360

**Published:** 2025-10-06

**Authors:** Mirko Vanzan, Susanna Bertuletti, Giacomo Becatti, Belen Bazan, Minze T. Rispens, Steven I. C. Wan, Michel Leeman, Willem L. Noorduin, Francesca Baletto

**Affiliations:** † Department of Physics, 9304 University of Milan, Via Celoria 16, 20133 Milan, Italy; ‡ 55952 AMOLF, Science Park 104, 1098 XG Amsterdam, The Netherlands; § 640285 Symeres, Kadijk 3, 9747 AT Groningen, The Netherlands; ∥ Van’t Hoff Institute for Molecular Sciences, University of Amsterdam, Science Park 904, 1090 GD Amsterdam, The Netherlands

## Abstract

Helicenes are a class of molecules potentially suitable in several technological applications with intrinsic structural chirality, and they are known for their exceptional chiroptical properties, with CD signals being notably more intense than those of other small organic molecules. Accurately estimating the chiroptical properties of helicenes is relevant for the application of these molecules in many diverse fields, yet still challenging. In this paper, we combine experimental optical characterization and *ab initio* calculations to study how different substituents influence the chiroptical properties of [6]­helicene. By systematically varying the size and chemical nature of the substituents, we find that both electron withdrawing and electron-donating substituents red-shift and dwindle the optical activity of the molecule. We hypothesize that the observed dumping in transition energy and intensity is connected to the strength of the perturbation induced by the substituent on the π-conjugation of the aromatic rings. The comparison between experiments and computations allows rationalization of the trends and suggestion of how the substituents influence properties. This work provides a framework for the fine-tuning of helicenes’ chiroptical properties via chemical modification of the substituents, enabling the design of helicene-based systems with tailored optical properties.

## Graphical abstract

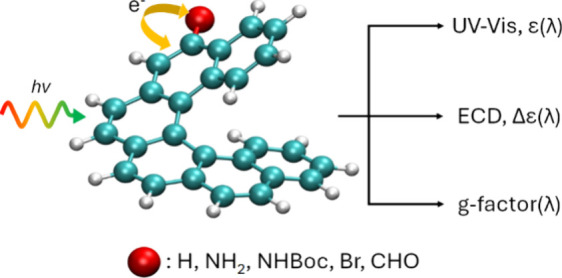

## Introduction

Helicenes are a class of organic compounds whose backbone consists of a series of ortho-fused phenyl rings.^1,2^ This configuration forces these molecules to deviate from the planar conformation usually adopted by aromatic compounds and to assume a helicoidal backbone. As the helicity of the backbone can be either left- or right-handed, these molecules exist in two different enantiomeric forms.

Ever since their discovery in the early 1900s, the organic chemistry community has devoted efforts into finding fast and effective methods to synthesize these compounds.^2–4^ Since the 1970s, it is possible to produce numerous and increasingly complex helicenes, due to the introduction of synthetic strategies such as Diels–Alder reactions, ring-closing metathesis, metal-catalyzed coupling and photoinduced cyclizations.^5–10^

Nowadays, helicene chemistry is extensively investigated as these molecular scaffolds are exploited in a highly diversified panel of fields such as metal complexes, molecular magnetism, nanorobotics, renewable energies and molecular photonics.^1,11–14^

Helicenes show unique optoelectronic properties, especially when interacting with polarized light, because of their intrinsic chirality.

To date, a theoretical model able to capture the molecular optical features starting from helicene’s structure (and *vice versa*) is still unavailable due to the variety and complexity that helicenes can present. However, being able to finely control the chiroptical response of these systems is of fundamental interest and represents a key step toward their efficient use in technologically relevant applications. From this perspective, *ab initio* simulations based on Time Dependent Density Functional Theory (TDDFT) represent a valuable tool to predict and optimize helicenes’ optoelectronic properties as they can reasonably reproduce the time-dependent optoelectronic response of molecular systems with moderate computational costs.^15^ A first seminal work in this sense comes from Furche and co-workers, who were the able to calculate [*n*]­helicenes electronic circular dichroism (ECD) spectra by means TDDFT in the early 2000s.^16^ Since then, the interest in the *ab initio* treatment of these compounds has progressively grown, and currently a vast amount of ECD spectra and g-factor (also called dissymmetry factor, a measure of the molecular chiroptical activity) estimates are available in the literature.^17–21^ Despite such progress, estimating the chiroptical properties of helicenes remains challenging because of the mutual interaction between the π-conjugated structure and the substituents. Achieving accurate predictions is essential for designing materials for cutting-edge applications in optoelectronics, sensing, and quantum devices, making this a crucial area of ongoing research.

In this work, we propose a link between [6]­helicenes’ chiroptical properties, intended as absorption, ECD, and g-factor spectra, and a substituent anchored to its backbone. To do that, we apply a hybrid approach, combining experimental and computational characterizations on a panel of derivatives of [6]­helicene. We focus on 5-monosubstituted molecules, where the substituent group is either an amino (NH_2_), a *tert*-butyloxycarbonyl (NHBoc), a bromide (Br) or a formyl (CHO). This pool of substituents differs both in size and in chemical nature, being the group either electron donor (as in the case of NH_2_), or withdrawing (like CHO). This diversity allows us to correlate the nature of the anchoring group to the optical response of the system. Figure 1 lists the investigated [6]­helicene derivatives and their corresponding abbreviations from the most activating (NH_2_) to the most deactivating (CHO) group from the point of view of electrophilic aromatic substitutions, as shown also by the Hammet constants σ_p_ in Table 1.

**Figure 1:**
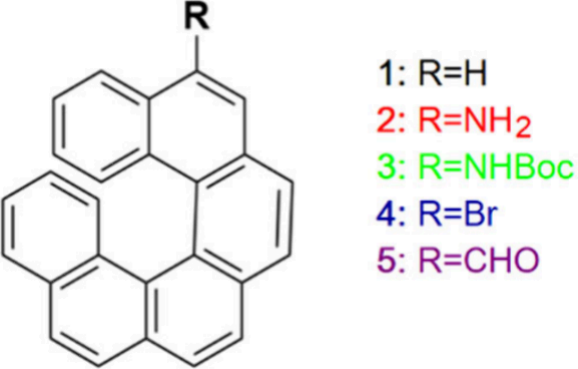
Schematic representation of the [6]­helicenes investigated in this work. The color code is used in Figures 2–5.

**Table 1:** Collection of the Molecular Properties^a^

Molecule	R	σ_p_	*Q*_H_	Δ*Q*_C–X_ ^a^	CCM	HCM
1	H	0	0^b^	0^b^	9.88	0.222
2	NH_2_	–0.66	–0.063	0.249	9.04	0.214
3	NHBoc	–0.05	–0.053	0.145	5.78	0.162
4	Br	0.23	–0.005	0.010	9.97	0.213
5	CHO	0.42	+0.091	0.110	9.18	0.208

a Substituents (R) Hammet constants (σ), helix backbone partial charges (*Q*_H_), C–X excess charge difference (Δ*Q*_C–X_), and CCM and HCM. Charges are given in [e] units. Negative and positive values indicate electron excess and depletion, respectively. σ_p_ values are taken from ref 50.

b C is the substituted carbon and X is the atom of R anchored to the backbone.

c Values set to zero as these quantities apply only to functionalized helicene.

We focus on the left-handed (*m*-)­enantiomers, with the substituent always bound to the same carbon atom, preventing eventual spurious comparisons connected to differences in the group’s relative position. Moreover, the chosen functional groups are all achiral, preventing the molecular chiroptical responses from being affected by the presence of further stereogenic centers.

Apart from bare [6]­helicene,^22–25^ the molecules selected for this study have been poorly investigated. To date, there is only a single publication reporting an ECD spectra for **2**,^26^ while no results are available in the case of **3** and **5**. Regarding **4**, there are a few references showing its importance in helicene chemistry and its optical activity, but they cannot be directly compared to our case as they refer to different isomers.^27–29^

Given the importance of helicenes, understanding how minor chemical modifications can impact their chiroptical properties and finding a physical rationale behind these changes is of outmost importance. In this view, this work aims to provide not only a new theoretical framework to interpret the optical properties of functionalized helicenes but also novel experimental and computational results on common, yet poorly studied, helicene compounds.

## Materials and Methods

### Synthesis

Chemicals were purchased from Merck, Ambeed and Fisher Scientific and used as such without further purification.

The syntheses of compounds **1**–**4** is reported in the Supporting Information, as they were previously described in the literature. The procedure to synthesize novel compound **5** is herein reported.

P/M-hexahelicene-1-carbaldehyde (**5**). Compound P-**4** (40 mg, 1 equiv, 98 μmol) was dissolved in dry THF (6.00 mL). The solution was cooled to <−78 °C (dry ice-acetone bath). *n*-BuLi (16 mg, 98 μL, 2.50 M, 2.50 equiv, 0.25 mmol) was added dropwise (*T* < −78 °C). The yellow reaction was stirred at −78 °C for 5 min. Dry DMF (0.14 g, 0.15 mL, 20 equiv, 2.0 mmol) was added dropwise (*T* < −78 °C). The reaction was continued at −78 °C for 2 h. The reaction was quenched by the dropwise addition of NH_4_Cl (aq, sat., 1 mL, *T* < −60 °C). The reaction was allowed to reach room temperature and was partitioned between water (10 mL) and DCM (10 mL). The water phase was extracted with DCM (2 × 10 mL). The combined organic phases were washed with water (10 mL) and brine (10 mL), dried over Na_2_SO_4_, filtered, and concentrated in vacuo to give a yellow solid. The material was submitted for preparative HPLC by dissolving it in a mixture of DMSO: THF (1:3, 2 mL) to give P-5 as a yellow solid (4 mg, 10%) with an enantiomeric purity of 97%.

^1^H NMR (400 MHz, CDCl_3_): δ 10.55 (s, 1H), 9.20 (ddd, *J* = 8.3, 1.3, 0.6 Hz, 1H), 8.49 (s, 1H), 8.10 (s, 2H), 8.09–8.01 (m, 2H), 7.95 (s, 2H), 7.83 (ddd, *J* = 8.0, 1.4, 0.6 Hz, 1H), 7.62 (dt, *J* = 8.5, 1.1 Hz, 1H), 7.50–7.45 (m, 1H), 7.34 (ddd, *J* = 8.3, 6.9, 1.3 Hz, 1H), 7.25–7.20 (m, 1H), 6.72 (dtd, *J* = 8.4, 6.9, 1.4 Hz, 2H).

### Characterization

CD spectra were measured with a Jasco J-1500 CD spectrometer. Samples were dissolved at 0.023 mg/mL in CH_2_Cl_2_ and analyzed in 10 mm quartz cuvettes (Hellma analytics QS – High precision).

Proton nuclear magnetic resonance (^1^H NMR) spectra were obtained on Bruker Avance Neo 400 MHz and Agilent VNMRS 300 MHz instruments. Chemical shifts for proton are reported in parts per million (ppm) (CDCl_3_, 7.26 ppm). The following designations are used to describe multiplicities: s (singlet), d (doublet), t (triplet), q (quartet), m (multiplet), br (broad). The obtained spectra were evaluated with the program MestReNova. All ^1^H NMR spectra are available as Supporting Information.

LC–MS spectra data were obtained with a Waters Acquity UPLC instrument with PDA detector and QDA mass detector system.

Column chromatography was carried out on a BUCHI flash chromatography system. All LC–MS spectra are available as Supporting Information.

Data coming from UV–vis absorption spectra in absorbance units were converted to molar extinction units through the Lambert–Beer equation. Concerning ECD, the instrument returns the solution ellipticity θ in millidegrees, which have been converted to Δε using the following relation:

1 $$\Delta \varepsilon =\frac{\theta \cdot MM}{C\cdot L\cdot 32980}$$

where Δε is in L·mol^–1^·cm^–1^, *C* is the experimental concentration of the sample in g·L^–1^, *L* is the length of the experimental optical path in cm and MM is the molar mass of the molecule in g·mol^–1^. In the case of molecules synthesized with a right-handed helicity, the experimental ECD data have been changed in sign to represent the ECD of the left-handed enantiomer. All data are obtained on dichloromethane solutions of newly synthesized molecules.

Considering all measures are taken at room temperature, we confidently exclude any racemization under analytical conditions, as this process starts to occur above 150 °C for these kind of molecules.^28^

### *Ab Initio* Simulations

Calculations were performed in a real-space domain, using the Octopus package version 12.2.^30–32^ We solved the ground-state electronic problem on a grid built with a 0.14 Å spacing between the points along each main Cartesian axes. The grid was contained within a box composed of the intersections of spheres of radius 3.5 Å, centered on every atom of the molecule. Such combination of input parameters ensures reaching reliable ground-state energies within a tolerance <10 meV and reliable optical properties up to 150 nm. We accounted for electron–electron interactions through the BP86 exchange–correlation (XC) functional,^33,34^ which has already been used in the literature to model helicenes in good agreement with experimental data.^16,35^ Given the peculiar shape of the investigated molecules, van der Waals interactions between different parts of the helices may play a role. We thus added a correction to the Hamiltonian, as described by Tkatchenko and Scheffler.^36^ Deep core states were modeled by standard Optimized Norm-Conserving Vanderbilt (ONCV) pseudopotentials. Geometrical structures of the molecules were optimized through the FIRE integrator^37^ setting a tolerance on the residual forces of 5 × 10^–3^ eV/Å. Optical calculations were performed in real-time^38,39^ through the δ-kick method. We used a kick strength of 0.1 V/nm, small enough to assume to be in a linear regime.^40^ The perturbation was applied along the three main Cartesian axes, and the results were averaged to obtain the isotropic molecular optical response. Time propagation was conducted using the exponential midpoint rule, with a time step size of 5 × 10^–4^ fs, reaching a total trajectory length of 10 fs. In the case of [6]­helicene, we also lengthen the simulation up to 20 fs to better resolve spectra without sensibly increasing the agreement with the experiments. All the calculations included the effect of the solvent as per the Integral Equation Formalism of Polarizable Continuum Model (IEF-PCM).^41–43^ To account for the presence of DCM as solvent, the static and dynamic dielectric constants were set to 9 and 2, respectively. To obtain ECD spectra we calculated the rotatory strength as implemented in the code through the oct-propagation_spectrum utility, starting from the real-time evolution of the electric dipole moment and orbital angular momentum, using a damping factor of 0.1 eV. We then converted the resulting rotatory strength and then calculated the difference in molar extinction coefficients Δε as reported in refs 44 and 45.

To obtain better agreement between theoretical and experimental data, the theoretical absorption and ECD spectra were systematically blue-shifted by 60 nm. It is known that BP86, like other Generalized Gradient Approximation XC functionals, provides an over-delocalization of electronic states, resulting in a red shift of the excitation energies.^46,47^ Moreover, our calculations included neither excitonic effects nor quasiparticle corrections, which could play a role in reproducing the optical spectra.

Formally, the discrepancy between computational and experimental data depends on the excitation energy itself; however, a 60 nm rigid shift turned out to be enough to cure such a discrepancy in the visible range. This procedure is commonly performed in the literature for molecular systems like the ones we investigated here.^19,25,48,49^

The accuracy of our results was further assessed using the CAM-B3LYP functional to account for possible charge-transfer phenomena between the helicene and its substituents. The results, detailed in the Supporting Information, show that CAM-B3LYP improved the prediction of excitation energies, but the overall shapes of the spectra are less accurate than those produced with BP86. Therefore, in the following we mainly discuss BP86 results in comparison with experimental data.

## Results and Discussion

The experimental and theoretical UV–vis absorption spectra of the molecules are shown in Figure 2a. All compounds present similar optical features, with a main absorption band in the range of 300–375 nm and a spectral fine structure, which depends on the specific molecular species, as visible from the experimental results (left side of Figure 2a). Compound **3** presents the most intense absorption peaks, followed by **1**, **4**, **2** and **5**. The intense absorption of molecule **3** can be related to its size, significantly larger than that of the other compounds, and to the presence of an amide bond, resulting in a larger absorption cross section and additional absorption in the 340–350 nm region.

**Figure 2:**
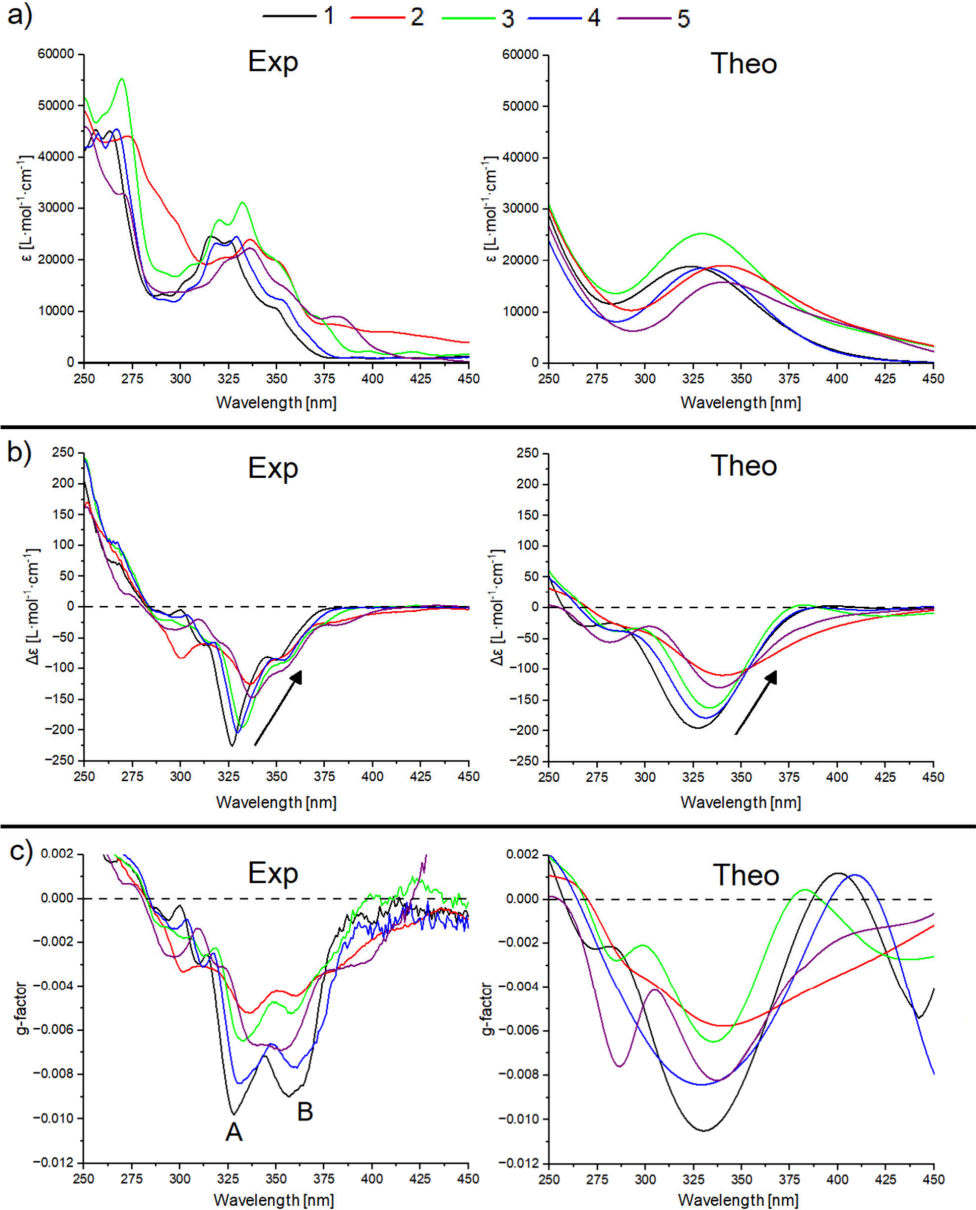
Collection of experimental (left side) and theoretical (right side) optical spectra and g-factors. a) UV–vis absorption spectra and b) ECD spectra, with inset arrows highlighting the red shift of the main peak. c) g-factor. A and B label the two main peaks. Theoretical results are blue-shifted by 60 nm. The color and numbering codes correspond to those used in Figure 1. One-by-one comparisons between experimental and theoretical results are available in the Supporting Information (Figures S1–S3).

Concerning the position of the experimental (theoretical) main absorption peaks, they fall at 316 (323) nm, 329 (330) nm, 332 (330) nm, 336 (339) nm, and 336 (340) nm for molecules **1**, **4**, **3**, **2** and **5**, respectively. This order reflects the magnitude of destabilization the substituent has on the aromatic rings. In fact, from an electronic point of view, Br (**4**), NHBoc (**3**), CHO (**5**) and NH_2_ (**2**) increasingly perturb the π-conjugation of the aromatic ring system.

The effect of the substituents’ nature on the electron redistribution within the π-conjugated system was analyzed by calculating the partial atomic charges at the ground-state Density Functional Theory (DFT) level. We define *Q*_H_ in the case of functionalized helicenes as the sum of all C and H atoms partial Hirshfeld charges^51,52^ not belonging to the substituent R, but constituting the helix backbone. As visible from Table 1, as the group provides more (positive or negative) charge on the aromatic rings, it accordingly affects the optical properties. Another common way to quantify π-perturbation in benzene-like aromatic systems is the substituents’ Hammet parameters for para electrophilic additions (σ_p_).^53^ Such a number provides a qualitative measure of the effect a group has on the aromatic system and directly correlates with *Q*_H_, as is visible from Table 1. Together with the molecules’ DFT-optimized geometries, partial atomic Hirshfeld charges are available in the Supporting Information.

To conclude the discussion on the absorption spectra, we notice that the most destabilizing groups (NH_2_ and CHO) give to the compounds a non-negligible absorption also at longer wavelengths, with molecule **2** absorbing light up to 500 nm. On the other hand, molecules **1** and **4** present a very similar absorption fingerprint, as Br weakly affects the electronic structure and thus the optical properties of the helicene scaffold, as confirmed by its small *Q*_H_ value (see Table 1). These observations represent a first direct proof of the influence that the substituents’ nature can have on the optical properties of helicenes. The considerations we made regarding the shapes of the absorption spectra apply also to the TDDFT calculations (Figure 2a, right side). As expected, the calculations do not reproduce the fine structure of the spectra, as they miss the vibrational contribution to the optical absorption. However, they show the same trends as the experiments, with peaks’ intensities underestimated of about 20–30%, an effect connected to the chosen computational setup.^54^

The experimental and theoretical ECD spectra of the investigated compounds are shown in Figure 2b. The ECD profiles show negative values because we studied the *m*-enantiomers of the compounds; *p*-enantiomers would present a mirrored trend with respect to the values of Δε (the difference in molar extinction coefficients for left- and right-handed circularly polarized light). Even in this case, all compounds present similar optical features, with a main band located within a smaller wavelength window (310–350 nm), and the fine structure of the spectrum depending on the specific molecular species.

Compounds **1**, **3** and **4** present sharper Δε bands compared to **2** and **5**, as visible from the experimental results (left side of Figure 2b). On the other hand, molecules **2** and **5** present a more sophisticated spectrum, with some peaks also around 300 nm. Compound **1** shows the most intense Δε peaks, followed by **4**, **3**, **1** and **2**. Generally, the weaker the peaks, the more red-shifted the wavelength of maximum Δε, as highlighted by the arrows in Figure 2b. The positions of the experimental (theoretical) main peaks indeed fall at 327 (327), 330 (332), 332 (333), 336 (340), and 337 (339) nm for molecules **1**, **4**, **3**, **2** and **5**, respectively. An exception is given by molecule **5**, which shows an ECD maximum at a wavelength slightly higher (by 1 nm) than that of **2**, although the peak intensity is higher. Nevertheless, the similarities between the UV–vis spectra of **1** and **4** still hold in the case of the ECD.

These findings are all consistent with the charge redistribution between the helicoidal scaffold and its substituent, as previously discussed. ECD indeed arises from chiral electronic transitions, which are directly influenced by the electronic density of the conjugated π-system. The higher the perturbation on the electronic structure of the aromatic rings, the lower and less energetic the chiral response of the compound is. This proves that the nature of the functional group also affects the chiroptical response of helicenes and in turn it confirms the well-known fact that it is possible to achieve fine-tuning of the molecular optical properties by chemical modification of a small portion of the molecule.^55–57^

The considerations we made on the shapes of ECD spectra also apply to the TDDFT calculations (Figure 2b, right side). Akin to calculated UV–Vis spectra, there is an underestimation of the peak’s intensity in calculated ECD spectra too; the theoretical peaks are 10–15% less intense than the experimental ones. However, the agreement between calculations and experiments is remarkable.

Experimental and computed spectra show the same relative trends of the ECD signal intensity for the library of compounds herein considered. Notably, both calculated absorption and ECD spectra of bare [6]­helicene (molecule **1**) can be directly compared with previous calculations made with similar computational setups, showing the consistency of our method.^16,22,25^

Absorption and ECD spectra can together yield information on the “absolute chirality” of a compound by means of the so-called g-factor or dissymmetry factor. This is a dimensionless number quantifying the asymmetry in the absorption of left- and right-handed circularly polarized light, and it is defined as the ratio between Δε and ε (i.e., ECD and UV–Vis spectra) as a function of the wavelength. Such a number is commonly adopted as a measure of the chiroptical activity of a compound and it is independent of the compound’s concentration in solution, hence allowing the direct comparison of different compounds in terms of “absolute chirality”.

Figure 2c shows the experimental and theoretical g-factors of the compounds. The experimental g-factor profiles clearly show a double peak shape, with the one falling at shorter wavelengths (peak A) being more intense than the one falling at longer wavelengths (peak B). The only exception is represented here by molecule **5**, where peak B is slightly more intense than peak A and the two-peak structure is barely visible.

The maximum absolute value of g-factors (|*g*_max_|) is on the order of 10^–2^–10^–3^, which lies within the typical value range for helicenes.^20^ The g-factor depends on the nature of the functional group, here following the trend **1**, **4**, **5**, **3**, **2**. Thus, bare [6]­helicene displays the highest g-factor among the compounds we studied here. Notably, the experimental |*g*_max_| falls in the same narrow wavelength window, regardless of the molecules. Specifically, peaks A (B) for |*g*_max_| fall at 328 (357) nm, 330 (361) nm, 338 (353) nm, 333 (357) nm, 336 (360) nm for molecules **1**, **4**, **5**, **3** and **2**, respectively, yet both A and B lie in a 10 nm range, indicating a stable peak profile.

We cannot provide any insights about the g-factor beyond 400 nm neither in the case of experimental measures nor in the case of calculated spectra since both Δε and ε tend to zero beyond 400 nm (see Figure 2a,b) and their ratios are unreliable.

The agreement between theoretical prediction and experiments holds in the case of calculated g-factor spectra too (Figure 2c, right side). Despite not capturing the double-peak motif, these closely follow the experimental trend with the main peak falling in the 330–340 nm range. We predicted an intensity 10–15% higher than the experimental one, due to the fact that our setup underestimates ε values^54^ and hence overestimates the g-factor peaks. Such a discrepancy in the intensity is more evident when ECD shows intense secondary peaks as in the case of molecule **5** (purple line in Figure 2c), which presents a strong yet unreliable peak also around 280 nm.

From our experimental and numerical data, it is possible to derive the dynamic polarizability tensor from the Kramers–Kroning relations. It is possible to find the real part of the chiral polarizability (α_c_), as well as the rest of the complete dynamic polarizability tensor, in our online open-access database.^58^

Here, we focus on the imaginary part of α_c_, i.e., the off-diagonal component of the dynamic polarizability tensor,^59^ simply converting the absorption signal in polarizability units.^25^ This quantity is of fundamental importance in the field of chiral nanophotonics and in recent years it has been playing an increasingly important role in applications exploiting optical forces as a way to control the dynamics of small particles.^60,61^

Figure 3 shows the wavelength dependence of the imaginary part of α_c_ extrapolated from the experimental (a) and computational (b) data. As visible from Figure 3a, all compounds present similar trends, with a main band in the range 310–350 nm and a fine structure depending on the molecular species.

**Figure 3:**
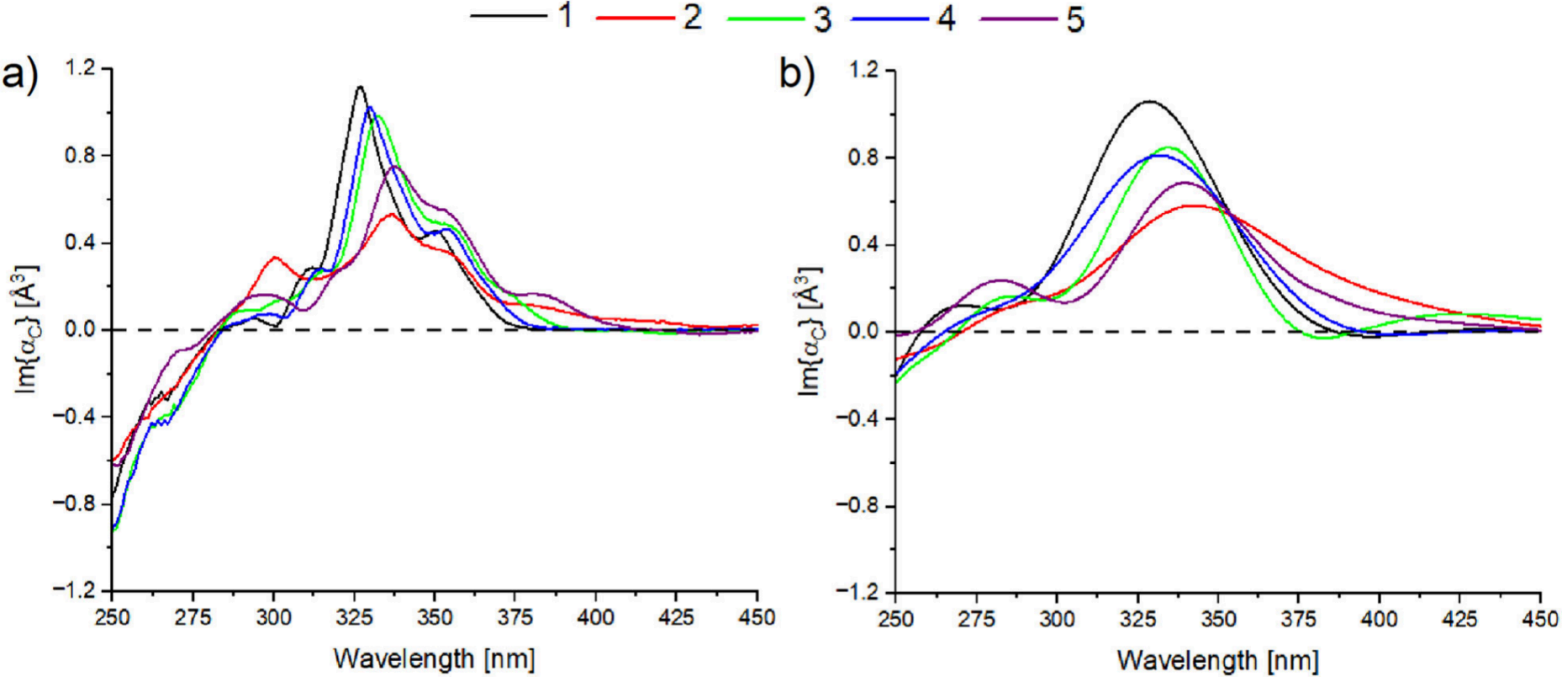
a) and b) show the estimated imaginary part of chiral polarizability for experimental and computational data, respectively. Theoretical results are red-shifted by 60 nm. The color and numbering codes correspond to those used in Figure 1. One-by-one comparisons between experimental and theoretical results are available in the Supporting Information (Figure S4).

Compound **1** presents the most intense peak, followed by **4**, **3**, **1** and **2** and weaker peaks translate into a more red-shifted wavelength of the maximum, which is consistent with the results from the ECD calculations (see Figure 2b).

These findings enforce the theory that the charge redistribution between the helicoidal scaffold and the substituent affects the optical responses. Chiral electronic transitions are naturally affected by the electronic density of the conjugated π-system, and the stronger the perturbation on the electronic structure, the lower and less energetic the chiral response of the compound is, as previously highlighted.

The considerations we made for Figure 3a also apply to Figure 3b. Compared to the experimental data, the underestimation of the peak’s intensity is around 10–15%, but calculations and experiments still show the same relative trends.

The g-factor depends on the chiroptical properties of the molecules, which in turn depend on both their geometry and their chemical features. To distinguish between these two effects, we analyzed how |*g*_max_| correlates with these aspects. We quantified the geometrical chirality of our molecules through the Continuous Chirality Measure (CCM)^62^ and the Hausdorff Chirality Measure (HCM).^63^ These descriptors are commonly used to measure the geometrical chirality of a system, and hence they can enable a fast screening of the chiroptical activity of molecular systems from their structure.

CCM was calculated throughout the web application in ref 64, while HCM was estimated using an in-house written code based on the procedure described in ref 65. CCM assigns a positive number to a structure, measuring the distance between the actual molecular configuration and the nearest achiral counterpart, with larger values indicating higher chirality degree. On the other hand, HCM quantifies the chirality of a system by representing the molecule as a collection of geometrical points and calculating the Hausdorff distance between the sets of the atomic positions and the set of the atomic position of the molecule’s enantiomer. More details on those methods are given in previous works.^63,66–68^

The dependence of |*g*_max_| on these measures is shown in Figure 4a,b. From the plots, it is clear how, in general, higher degrees of geometrical chirality provide higher |*g*_max_|, from both an experimental and a theoretical point of view. However, it is difficult to assess a direct proportionality between these quantities as there are exceptions to this rule. For example, despite showing a larger CCM, molecules **2** and **4** provide weaker |*g*_max_| than **3** and **1**, respectively. For the sake of completeness, consider the CCM of bare [6]­helicene (**1**) is 9.88 and this value is compatible with previous estimations.^69^ Similar considerations hold in the case of HCM, where compound **4** yields the weakest |*g*_max_| despite showing the second-highest value in the HCM ranking (see also Table 1). Interestingly, the compound with the largest achiral substituent (NHBoc, molecule **3**) presents the lowest CCM and HCM, reflecting the capability of these methods to capture the structural chirality, regardless of the molecular optical activity. Although based on different mathematical frameworks,^70,71^ we found that CCM and HCM validate each other’s predictions. A correlation between those descriptors is available in the Supporting Information. Table 1 reports the CCM and HCM values for all of the considered molecules.

**Figure 4:**
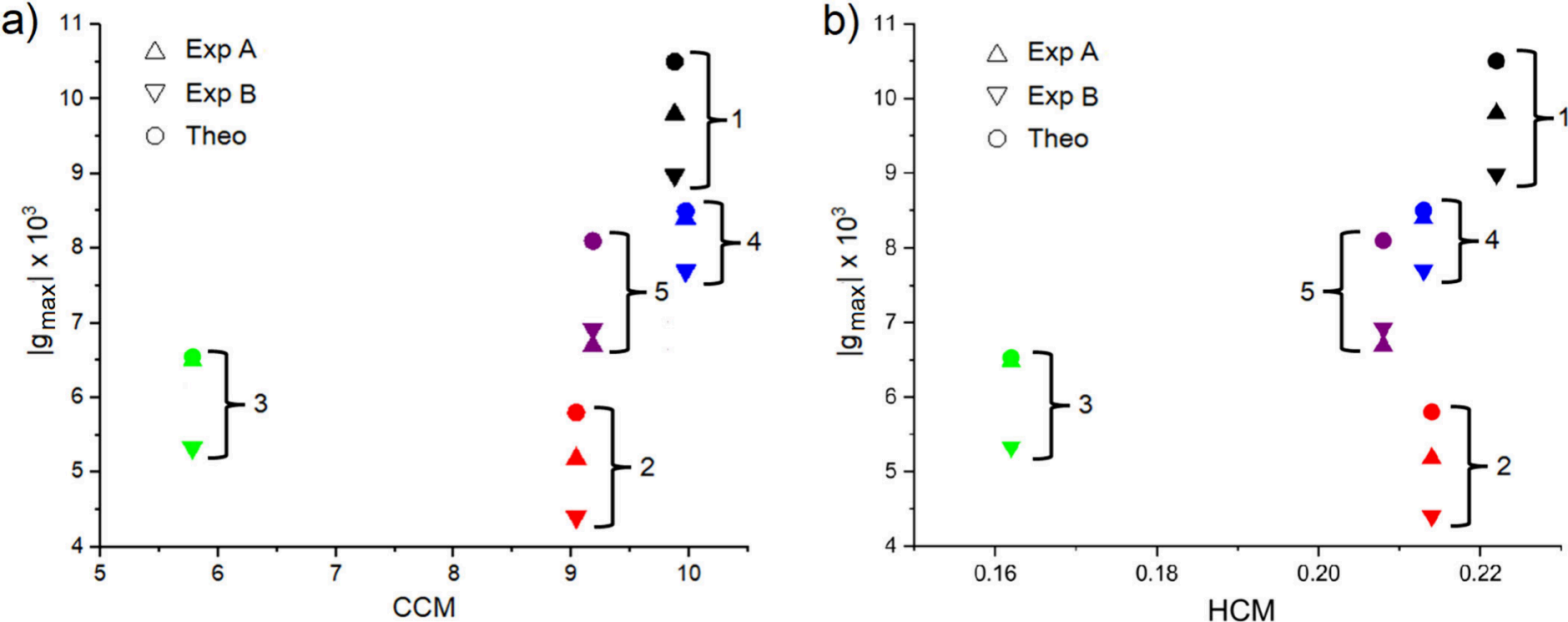
a) and b) show the correlation between the module of g-factor peaks’ intensities as a function of the CCM and HCM. Exp A and Exp B refer to the experimental peaks of the g-factor (see Figure 2c). Symbols color code given accordingly to the legend of Figure 2, and inset numbers label the molecule as per Figure 1.

Although the size of our compound library does not allow us to draw definitive conclusions, our analysis suggests that using achiral functional groups can quench the chirality of the whole structure, and the reduction could depend on the size of the group. Substituent size is therefore a key parameter when dealing with fine-tuning of the optical properties of helicenes. Taking a step forward, this also suggests that accounting for the same group size, functionalizing a helicene scaffold with a substituent presenting opposite chirality would reduce even more the systems’ chiroptical response. To date, we have no direct proof of this; however, such a hint may be the subject of future studies.

Figure 5a shows the relationship between experimental and theoretical |*g*_max_| and *Q*_H_, which resembles a volcano plot. Notice that *Q*_H_ for molecule **1** is set to zero to provide a reference point for relative comparison, as the measure of *Q*_H_ applies only in the case of functionalized helicenes. The maximum value of |*g*_max_| is obtained for [6]­helicene, i.e., where there are no heteroatoms perturbing the aromatic π-conjugation. Regardless of the nature of the functional group, we always notice a decrease in |*g*_max_| with the magnitude of this reduction depending on the amount of charge added to or subtracted from the helicene scaffold.

**Figure 5:**
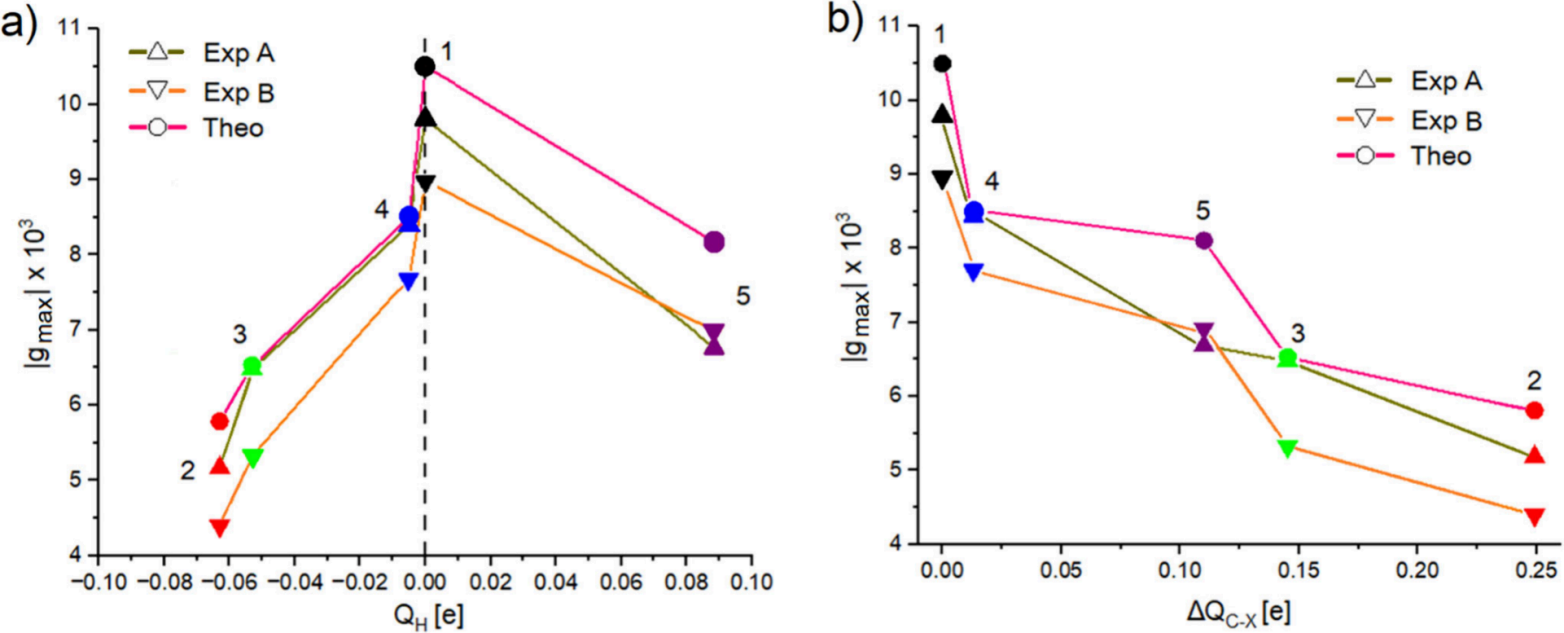
a) and b) show the correlation between the module of g-factor peaks’ intensities as a function of *Q*_H_ and Δ*Q*_C–X_ respectively. Exp A and Exp B refer to the experimental peaks of the g-factor (see Figure 2c). Symbols color code given accordingly to the legend of Figure 2, and inset numbers label the molecule as per Figure 1.

Our analysis suggests that electron donating groups tend to reduce both experimental and theoretical optical activities more than electron withdrawing substituents, as the slopes of the two edges of the volcano plot are clearly different. However, it is necessary to underline that this is a qualitative trend and is by no means intended to be a quantitative measure of the substituents’ effect. Indeed, we have only one case where *Q*_H_ is positive, and this is insufficient to draw statistically meaningful conclusions. In any case, this qualitative observation indicates that the C–X bond polarization (where X is the anchoring atom of the substituent group R) can affect the chiroptical properties of compounds. We therefore calculated Δ*Q*_C–X_, defined as the module of difference between partial C and X atomic charges. The higher this number, the more polarized the bond is. Precise values are in Table 1.

Figure 5b plots the correlation between experimental and theoretical |*g*_max_| and Δ*Q*_C–X_, showing that larger C–X bond polarizations are connected to lower chiroptical activity because of the larger perturbation induced on the aromatic π-conjugation.

This implies that a local electrostatic perturbation induced by the bond with the substituent can significantly change the physical properties of the helix. Even though there exist studies on the influence that various functional groups have on helicenes’ nonlinear optical features,^48,72^ a direct relation between helicenes’ chiroptical properties and local bond polarization has never been previously observed. By choice, we based our discussion on a ground-state electronic structure analysis. Investigating the excited-state electronic dynamics could uncover additional insights and may help us to understand how the substituent influences the chiroptical properties and mediates charge-transfer phenomena. We attempted to extract further information by analyzing the orbitals involved in the main optical transitions and the induced charge density, but the results were inconclusive (see the Supporting Information).

The limited set of molecules and functional groups examined restricts the applicability of our model to more complex systems, particularly those involving components with pronounced absorption features, such as metal ions or chromophores. Therefore, our theoretical framework is not universally applicable, and its extension to other helicenes should be approached with caution. Nonetheless, within its defined scope, our approach provides a simple and effective way to obtain a structure-optical relationship in helicenes bearing small organic substituents, enabling a fast screening of chiroptical activity and guiding a rational design toward molecules with tailored optical properties.

## Conclusion

This study highlights the critical role of substituent effects in modulating the chiroptical properties of [6]­helicene. Through the combined use of experimental measurements and *ab initio* simulations, we demonstrated that large achiral substituents decrease the geometrical chirality of the molecular structure and reduce its g-factor. Such a correlation was validated against two different chirality measures, highlighting a robust link between the substituent geometrical structures and the chiroptical response.

Additionally, we found that the chemical nature of the functional group remarkably affects the optical response of [6]­helicene. In particular, within the pool of explored molecules, the stronger the perturbation induced on the aromatic π-conjugation—both in terms of absolute charges localized on the rings and of carbon-substituent bond polarization—the more red-shifted the optically active transitions are, along with a dwindling of the ECD response and *g*-factor.

We demonstrated that a proper design of the length and shape of the functional group can enable precise control of the optical response of the systems, in terms of both absorption and ECD. Additionally, we proposed a simple yet effective method to estimate the influence of a substituent on helicene chiroptical properties, using its partial charge and the polarization of the substituent–helix bond as key descriptors.

Our findings advance the understanding of chiroptical properties of [6]­helicenes by offering new experimental and computational insights on either newly synthesized or previously underexplored derivatives. Moreover, our study increases the understanding of the structure–property relationships in helicenes, also providing a practical framework for tailoring their optical response through precise chemical modifications.

We stress that the suggested approach is not meant to provide a universal framework valid for all possible functional groups. We do not offer an in-depth investigation of the electronic structure or excited-state properties of the molecules, topics that warrant a dedicated study. Our numerical simulations aim to rationalize the observed trends and to suggest how the substituents can influence the chiroptical properties of [6]­helicenes. We showed that chiroptical properties can be forecasted from ground-state parameters, such as molecular symmetry and charge redistribution, that can be obtained through simple calculations or even qualitatively estimated. Based on those features, promising strategies to enhance g-factors may include varying the helicene substituent anchoring atom, using multiple substituents, or employing small strongly optically active groups that extend the π-conjugation with the backbone. Despite our limited data set, the proposed model proved to be robust in capturing trends in chiroptical properties.

Although real-time TDDFT has not been previously applied to this class of helicenes, the novelty of our work does not lie primarily in the methodology itself. Our work provides a scientific rationale for fine-tuning of helicenes’ chiroptical properties via chemical control of the substituents, thereby offering new and valuable insights into the rational design of chiral molecular systems with customizable optical properties for advanced photonic and optoelectronic applications.

## Supplementary Material



## Data Availability

Experimental and theoretical UV–vis, ECD and g-factor spectra are available at the following link: 10.13130/RD_UNIMI/XQWOE3. Moreover, all results are publicly available on our online database at https://chiraldb.fisica.unimi.it/

## Notes

Details on molecules synthesis; ^1^H NMR spectra; one-by-one comparison between experimental and computational results; correlation between CCM and HCM; considerations about BP86 and CAM-B3LYP XC functionals; molecular orbital analysis; induced charge density analysis; DFT-optimized structures and partial atomic Hirshfeld charges of the investigated molecules (PDF)
