# Supplementary material for: A Rational Framework to Estimate the Chiroptical Activity of [6]Helicene Derivatives
Source: J Phys Chem A. 2025 Oct 6;129(41):9537–47. doi: 10.1021/acs.jpca.5c04360 (PMC12536393; doi:10.1021/acs.jpca.5c04360)
Supplement: Supplementary file 1 [file jp5c04360_si_001.pdf]

Supporting Information

A Rational Framework to Estimate the Chiroptical Activity of [6]Helicene Derivatives

Mirko Vanzan[a,‡], Susanna Bertuletti[b,‡], Giacomo Becatti [a], Belen Bazan[c], Minze T. Rispens[c], Steven I.C. Wan[c], Michel Leeman[c], Willem L. Noorduin[b,d], Francesca Baletto[a,*]

*Corresponding author: francesca.baletto@unimi.it ‡These authors share the first authorship

a) Department of Physics, University of Milan, Via Celoria 16, 20133, Milan, Italy.
b) AMOLF, Science Park 104, 1098 XG, Amsterdam, The Netherlands.
c) Symeres, 9747 AT Groningen, The Netherlands.
d) Van't Hoff Institute for Molecular Sciences, University of Amsterdam, Science Park 904, Amsterdam, The Netherlands.

DETAILS ON THE SYNTHESIS

Materials

Chemicals were purchased from Merck, Ambeed and Fisher Scientific and used as such without further purification.

Synthesis

The synthesis of helicenes **2** and **3** starts with the synthesis of the two building blocks **8** and **14**. The synthesis of these building blocks are shown in Scheme 1 and Scheme 2. The coupling of the two building blocks and subsequent chemistry towards compounds **2** and **3** is shown in Scheme 3. The synthesis of helicenes **1**, **4** and **5** are shown in Scheme 4.

Synthesis towards building block 8

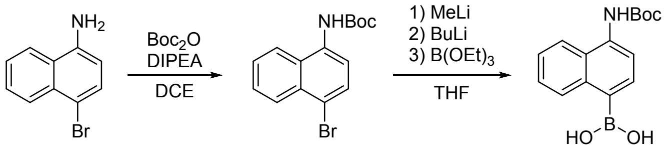

6 **7** **8**

Scheme 1: Synthesis towards intermediate **8**

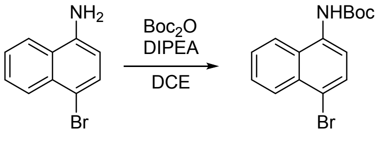

6 **7**

***tert*-Butyl (4-bromonaphthalen-1-yl)carbamate (7).** To a 2 L three-neck round bottom flask equipped with a reflux condenser, **6** (30.26 g, 136.3 mmol, 1.0 eq), di-*tert*-butyl dicarbonate (170.0 g, 778.9 mmol, 5.72 mmol), 1,2-dichloroethane (600 mL), *N*-ethyl-*N*-isopropylpropan-2-amine (52 mL, 300 mmol, 2.2 eq) were added. The mixture was heated to reflux overnight. The reaction mixture was check by TLC against 1 (1:4 EtOAc/heptanes, $KMnO_4$ stain, R_f: **6** = 0.29, **7** = 0.57), and full consumption of **6** was observed. The reaction was cooled to rt, then transferred to a 1L separatory funnel. The organic layer was washed with 1.2M HCl (3 x 100 mL), saturated sodium bicarbonate solution (100 mL) (pH = 8), then water (100 mL) and brine (100 mL),

where water (100 mL) was added to aid phase separation. The organic layer was then dried over Na_2SO_4, filtered into a 1L round bottom flask, and concentrated *in vacuo*. A large amount of reddish liquid was obtained, in which solid formed upon standing. The crude was recrystallized from heptanes, and further cooled on ice for 30 min after cooling down to rt. The crystals were collected via vacuum filtration, dried on filter and transferred to a tared crystallizing dish. After drying in the oven, then overnight at rt, 7 was isolated as an off-white solid (35.86 g, 82% yield).

^{1}H NMR (400 MHz, $CDCl_3$) δ 8.26 (dd, *J* = 7.8, 1.6 Hz, 1H), 7.87 (dd, *J* = 8.0, 1.6 Hz, 1H), 7.76 (q, *J* = 8.2 Hz, 2H), 7.59 (dqd, *J* = 8.2, 6.8, 1.4 Hz, 2H), 6.84 (s, 1H), 1.56 (s, 10H). Peak at 1.56 ppm shows 10H due to cumulative relaxation time difference.

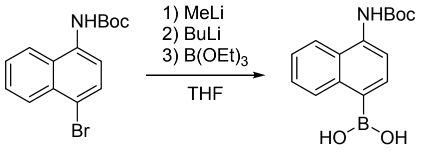

7 **8**

(4-((*tert*-Butoxycarbonyl)amino)naphthalen-1-yl)boronic acid (8). All glassware in contact with reaction mixture was dried in the oven overnight. To a 500 mL three-neck round bottom flask equipped with a mechanical stirrer under nitrogen atmosphere, **7** (14.9 g, 46.2 mmol, 1.0 eq) and anhydrous THF (95 mL) were added, resulting in a yellow homogeneous solution. The reaction was cooled on a salt-ice bath until 0 °C or below. Methyllithium (1.6 M solution in Et_2O, 28.8 mL, 46.1 mmol, 0.996 eq) was added dropwise, while keeping the temperature <7 °C. During addition the solution turned pale green, then golden yellow. After stirring for another 15 minutes, the solution was cooled in an acetone/dry ice bath until ~-70 °C. Butyllithium (2.5 M in hexane, 18.2 mL, 45.5 mmol, 0.984 eq) was added dropwise while keeping the temperature below -64 °C, upon which solution turned cloudy and green. After stirring for another 1h, triethyl borate (20.0 mL, 120 mmol, 2.5 eq) was added dropwise while keeping the temperature below -65 °C. The reaction mixture was gelating slowly during the addition. The solution was stirred for another 45 min at -65 °C, then 1.5 h at 0 °C. While at 0 °C, the reaction was quenched with dropwise addition of 1M HCl (125 mL). Precipitate appeared and clumped up. The pH was constantly monitored, and when the mixture reached pH 1, the reaction was stirred for an additional 15 minutes. The reaction mixture (except the precipitate) was transferred to a 1L separatory funnel and was saturated with NaCl. Et_2O (65 mL) was added to aid phase separation. The phases were separated, and the organic layer was extracted with 1M NaOH (4 x 125 mL). The combined basic aqueous layer was cooled on ice (with stirring) and acidify with 12M HCl dropwise until a persistent cloud of precipitate formed (pH 8). The aqueous layer was further acidified with 2M HCl until pH 1, then immediately extracted with Et_2O (3 x 150 mL) as the compound is not very stable towards acidic conditions. The combined organic layers were washed with water (100 mL, pH of aq layer = 6-7), brine (100 mL), dried over Na_2SO_4, and concentrated *in vacuo* (40 °C water bath) to yield **8** as a white solid as product (11.03 g, 83% yield)

^{1}H NMR (400 MHz, DMSO) δ 9.21 (s, 1H), 8.48 – 8.38 (m, 1H), 8.26 (s, 2H), 8.09 – 8.01 (m, 1H), 7.70 (d, *J* = 7.5 Hz, 1H), 7.54 (d, *J* = 7.5 Hz, 1H), 7.52 – 7.42 (m, 2H), 1.49 (s, 10H). Peak at 1.49 ppm shows 10H due to cumulative relaxation time difference.

Synthesis towards building block 14

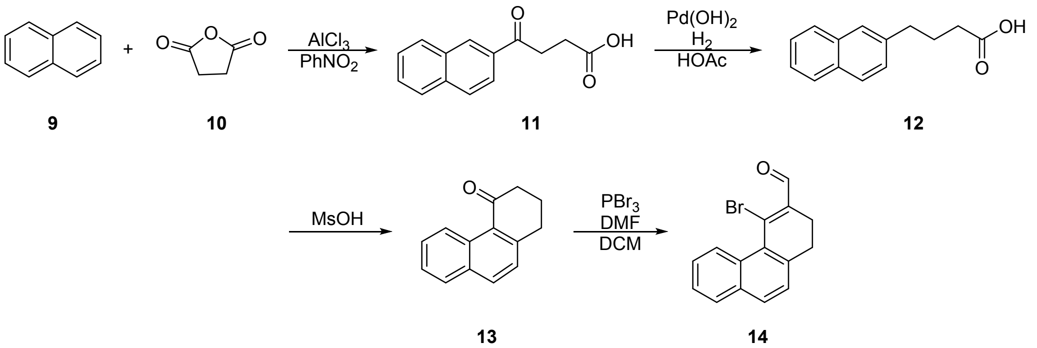

Scheme 2: Synthesis towards intermediate **14**

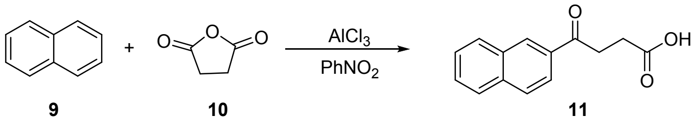

4-(Naphthalen-2-yl)-4-oxobutanoic acid (11). To a 2 L three-neck round bottom flask, nitrobenzene (400 mL) and aluminum trichloride (133.99 g, 1.005 mol, 2.00 eq) were added. Exotherm occurred as solid dissolved. After cooling back to rt, naphthalene (**9**, 96.39 g, 752 mmol, 1.49 eq) was added, where solution turned black, and an endotherm was noted. Succinic anhydride (**10**, 50.36 g, 503 mmol, 1.0 eq) was added, and an exotherm was noted as solid dissolved. After stirring for overnight, the mixture was poured into 1 L ice-water and 37% w/w HCl (100 mL) was added. Yellow precipitate was formed, and the mixture was stirred for 30 min. The precipitate was collected by vacuum filtration, and the precipitate was washed with water (200 mL) and heptanes (200 mL). The crude was suspended in toluene (450 mL) in a 1 L three-neck RBF equipped with a thermometer. After heating at 60 ^{o}C for 1h, the solution was cooled to 45 ^{o}C, and the precipitate was immediately collected via vacuum filtration, and washed with toluene (50 mL, rt). The solid was transferred to a beaker and dried in an oven until a constant weight was obtained. Pure **11** was obtained as an off-white powder (59.17 g, 51% yield).

^{1}H NMR (400 MHz, DMSO) δ 12.17 (s, 1H), 8.71 (d, *J* = 1.6 Hz, 1H), 8.15 (dd, *J* = 8.0, 1.5 Hz, 1H), 8.05 – 7.97 (m, 3H), 7.65 (dddd, *J* = 19.0, 8.2, 6.8, 1.4 Hz, 2H), 3.40 (dd, *J* = 6.8, 5.8 Hz, 2H), 2.65 (dd, *J* = 6.8, 5.9 Hz, 2H).

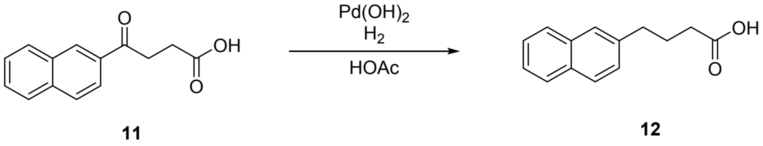

4-(Naphthalen-2-yl)butanoic acid (12). Acid **11** (25.07 g , 109.8 mmol, 1.0 eq), $Pd(OH)_2$ (6.41 g, 12% w/w, 5.48 mmol, 0.050 eq, ACROS brand, unreduced, 20% Pd, 60% moisture.) and acetic acid (230 mL) were added to a 500 mL three-neck RBF. Flask was equipped with hydrogen balloon, and the system was flushed with H_2. The mixture was stirred for 2 days at rt, during which the reaction was monitored by TLC (1:1 EtOAc/heptanes 5 mL + 3 drops AcOH, $KMnO_4$ stain, R_f: **11** = 0.4, **12** = 0.5). The reaction was filtered through a cake of wet celite and washed with AcOH. The slightly brown filtrate was poured into 1.5 L ice-water mixture, upon which white precipitate appeared. After stirring for 30 minutes, the solid was collected via vacuum filtration (glass funnel, por 3). The wet precipitate was scrapped off from the filter into a 1L Erlenmeyer flask, dissolved in EtOAc (400 mL), and transferred to a separatory funnel. Layers were separated, and the aqueous layer was extracted with EtOAc (100 mL). The combined organic layer was dried over Na_2SO_4, filtered and concentrated *in vacuo*. The resulting white solid was recrystallized from heptanes. The crystals were collected by vacuum filtration and washed with heptanes. The crystals were transferred to a tared recrystallization dish, and crush until constant weighed. 7 (17.67 g, 75% yield) was obtained as slightly pink crystals.

^{1}H NMR (400 MHz, $CDCl_3$) δ 7.93 – 7.74 (m, 3H), 7.65 (d, *J* = 1.8 Hz, 1H), 7.46 (pd, *J* = 6.9, 1.6 Hz, 2H), 7.35 (dd, *J* = 8.4, 1.7 Hz, 1H), 2.86 (t, *J* = 7.5 Hz, 2H), 2.43 (t, *J* = 7.4 Hz, 2H), 2.09 (p, *J* = 7.5 Hz, 2H).

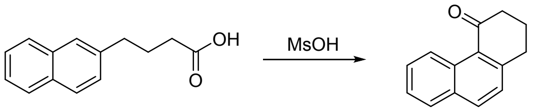

12 **13**

2,3-Dihydrophenanthren-4(1H)-one (13). To a 500 mL three-neck round bottom flask equipped with an air condenser and a thermometer, acid **12** (17.47 g, 81.5 mmol, 1.0 eq) and methanesulfonic acid (100 mL, 1.54 mol, 18.9 eq) were added. The mixture was heated to 85 °C (kept below 95 °C to avoid side product formation), where the initial inhomogeneous solution turned brown and homogeneous. After 1h, the solution turns green, and TLC (1:1 EtOAc/heptanes 5 mL + 3 drops AcOH, $KMnO_4$ stain, R_f: **12** = 0.5, **13** = 0.7.) indicated complete consumption of acid 7.[b] The reaction mixture was cooled to rt, poured into water (400 mL), and transferred to a 1L separatory funnel. The aqueous layer was extracted with MTBE (3 x 200 mL). The combined organic layer was washed with water (100 mL), saturated sodium bicarbonate solution (100 mL), then water (100 mL), then brine (100 mL), and dried over sodium sulfate, filtered into a 500 mL round bottom flask, and concentrated *in vacuo* to obtain **13** as a brown oil which solidified on standing (15.81 g, 99% yield).

^{1}H NMR (400 MHz, $CDCl_3$) δ 9.41 (dd, *J* = 8.5, 1.1 Hz, 1H), 7.93 (d, *J* = 8.3 Hz, 1H), 7.81 (dd, *J* = 8.1, 1.5 Hz, 1H), 7.63 (ddd, *J* = 8.7, 6.8, 1.5 Hz, 1H), 7.49 (ddd, *J* = 8.0, 6.8, 1.2 Hz, 1H), 7.33 (d, *J* = 8.4 Hz, 1H), 3.13 (t, *J* = 6.1 Hz, 2H), 2.79 (dd, *J* = 7.3, 6.0 Hz, 2H), 2.45 – 2.11 (m, 2H).

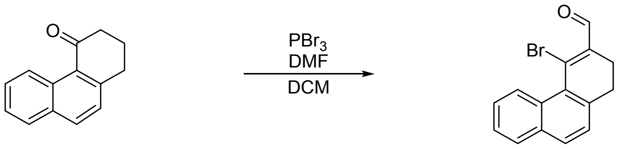

13 **14**

4-Bromo-1,2-dihydrophenanthrene-3-carbaldehyde (14). All glassware used were dried in oven for >1h at 105°C. Dry DCM was prepared by passing DCM through a short plug of silica.

To a 100 mL three-neck round bottom flask equipped with a reflux condenser under nitrogen atmosphere, dry DCM (50 mL) and dry DMF (4.7 mL, 61 mmol, 11 eq) were added. The mixture was cooled to 0 °C on a water-ice bath, and phosphorus tribromide (4.8 mL, 51 mmol, 9.5 eq) was added dropwise over 3 min, at which the solution turned slightly yellow and cloudy. After stirring the mixture at 0°C for 1h, ketone **13** (1.056 g, 5.38 mmol, 1.0 eq) was added to the mixture in one go. Solution turned red, and the cold water was swapped for a sand bath, and the solution was heated to reflux overnight (~18h). TLC (1:9 EtOAc/heptanes, $KMnO_4$ stain, R_f: **13** = 0.42, **14** = 0.56) indicate that starting material has been completely consumed. The reaction was subsequently cooled to rt, and the mixture was furthered cooled on an ice-water bath. NaOH solution (30% w/w) was added dropwise (some delay until the quenching of unreacted reagent proceeded) until no more fuming was observed. The solution was diluted with DCM (40 mL) and water (100 mL), transferred to a 500 mL separatory funnel, and the layers were separated. The aqueous layer was extracted with DCM (2 x 30 mL), and the combined organic layer was washed with water (2 x 30 mL) and dried over sodium sulfate, filtered into a 250 mL round bottom flask, and concentrated to yield a brown oil as crude product. The crude product was dissolved in EtOAc, coated onto celite, and purified by column chromatography (1:19 MTBE/heptane). Fractions were checked by TLC (1:9 EtOAc/heptane, $KMnO_4$ stain), and fractions with R_f~0.5 were combined and concentrated to yield a brown oil which crystallizes upon standing (1.029 g, 67% yield). Product must be stored in fridge under a blanket of nitrogen.

^{1}H NMR (400 MHz, $CDCl_3$) δ 10.32 (s, 1H), 8.71 (dd, *J* = 8.5, 1.1 Hz, 1H), 7.97 – 7.70 (m, 2H), 7.57 (ddd, *J* = 8.6, 6.9, 1.6 Hz, 1H), 7.50 (ddd, *J* = 8.1, 6.8, 1.2 Hz, 1H), 7.34 (d, *J* = 8.2 Hz, 1H), 2.92 – 2.80 (m, 2H), 2.62 – 2.50 (m, 2H).

Synthesis towards helicenes 2 and 3

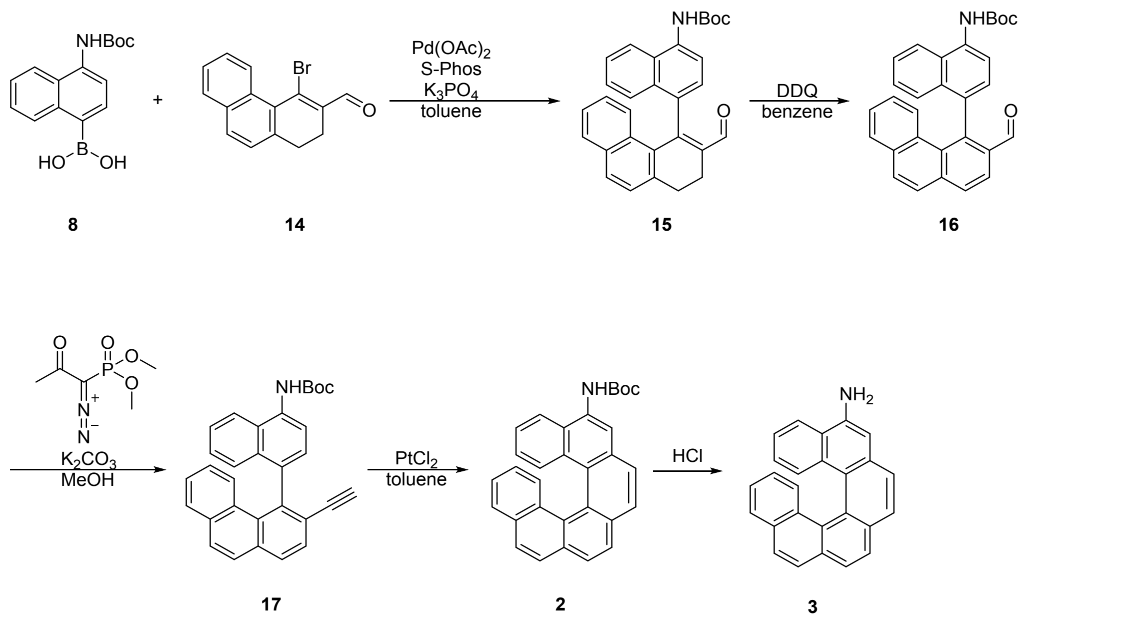

Scheme 3: Synthesis towards helicenes **2** and **3**

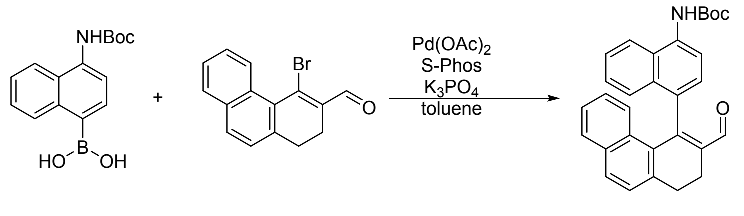

8 **14** **15**

***tert*-Butyl (4-(3-formyl-1,2-dihydrophenanthren-4-yl)naphthalen-1-yl)carbamate (15).** To a 250 mL three-neck round bottom flask equipped with an air condenser under nitrogen atmosphere, **8** (6.373 g, 22.20 mmol, 1.50 eq), 2-Dicyclohexylphosphino-2',6'-dimethoxybiphenyl (161 mg, 0.392 mmol, 0.026 eq), palladium (II) acetate (36.8 mg, 0.164 mmol, 0.011 eq), K_3PO_4 (6.40 g, 30.2 mmol, 2.03 eq), and **14** (4.258 g, 14.83 mmol, 1.0 eq), degassed toluene (87 mL),[a] water (0.13 mL) were added at room temperature in that order. The mixture was heated (heating unit = 110°C) until a gentle boiling was observed. After 4h, LCMS indicates complete consumption of **14**, and the reaction was cooled to rt. The reaction was then filtered through a cotton plug with vacuum suction. The black residue was washed with EtOAc (40 mL) and water (40 mL). The filtrate was transferred to a 500 mL separatory funnel and washed with water (80 mL). The aqueous layer

was subsequently extracted with EtOAc (2 x 50 mL), and the combined organic layer was washed with water (2x 100 mL) and brine (100 mL), dried over Na_2SO_4 (overnight stirring), filtered and concentrated *in vacuo* to yield yellows flakes as crude. The crude was dissolved in EtOAc and coated onto celite, loaded onto a silica column for column chromatography (1:19 EtOAc/heptanes, ~2CV, then 1:9 EtOAc/heptanes, then eventually EtOAc). Fractions were checked with TLC (1:4 EtOAc/heptane), and fractions with R_f ~ 0.4 were combined and concentrated *in vacuo* to yield a yellow powder as product (6.60 g, quantitative yield), but contains a few minor peaks in ^{1}H-NMR as impurities.

^{1}H NMR (400 MHz, $CDCl_3$) δ 9.52 (s, 1H), 8.07 (d, *J* = 7.8 Hz, 1H), 7.90 (d, *J* = 8.5 Hz, 1H), 7.82 (d, *J* = 8.2 Hz, 1H), 7.71 – 7.66 (m, 1H), 7.63 – 7.56 (m, 1H), 7.50 – 7.44 (m, 3H), 7.30 – 7.27 (m, 1H), 7.22 – 7.13 (m, 2H), 7.02 (s, 1H), 6.80 (ddd, *J* = 8.9, 7.0, 1.5 Hz, 1H), 3.16 – 2.98 (m, 2H), 2.86 (dt, *J* = 14.4, 7.0 Hz, 1H), 2.69 (ddd, *J* = 15.0, 9.4, 7.5 Hz, 1H), 1.59 (s, 9H).

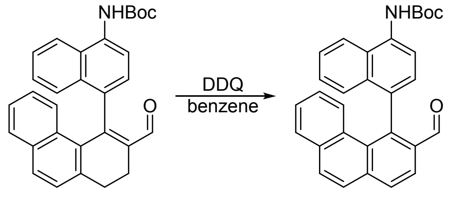

15 **16**

***tert*-Butyl (4-(3-formylphenanthren-4-yl)naphthalen-1-yl)carbamate (16).** To a 1L three-neck round bottom flask equipped with a reflux condenser, **15** (6.70 g, 14.9 mmol, 1.0 eq), DDQ (5.42 g, 23.9 mmol, 1.60 eq) and benzene (435 mL) were added. The resulting dark red mixture was heated to reflux for 29h, upon which full consumption of **15** was observed by LCMS. The reaction mixture was then cool to rt, filtered through a cotton plug with vacuum suction into a 1L round bottom flask. The residue was rinsed with toluene, and the combined filtrate was transferred to a 1L separatory funnel. The organic layer was washed with 1M NaOH (3 x 330 mL), then the combined aqueous layer was extracted with EtOAc (250 mL) and combined with the previous organic layer. The combined organic layer was washed with water (100 mL) and brine (100 mL), dried over Na_2SO_4, filtered and concentrated *in vacuo* to yield a dark green wax. The wax was then dissolved in a minimum amount of EtOAc, mixed with an approximately equal amount of heptanes, and concentrated *in vacuo* to yield **16** as a yellow-green powder (6.62 g, quantitative yield).

^{1}H NMR (400 MHz, $CDCl_3$) δ 9.46 (s, 1H), 8.26 (d, *J* = 8.3 Hz, 1H), 8.18 (d, *J* = 7.7 Hz, 1H), 8.09 (d, *J* = 8.3 Hz, 1H), 8.03 (d, *J* = 8.6 Hz, 1H), 7.90 (d, *J* = 8.8 Hz, 1H), 7.86 (d, *J* = 8.9 Hz, 1H), 7.82 (dd, *J* = 7.9, 1.5 Hz, 1H), 7.55 (ddd, *J* = 8.4, 6.7, 1.4 Hz, 1H), 7.49 (d, *J* = 7.8 Hz, 1H), 7.37 (t, *J* = 6.9 Hz, 2H), 7.34 – 7.27 (m, 2H), 7.11 (s, 1H), 6.92 (ddd, *J* = 8.7, 7.0, 1.6 Hz, 1H), 1.62 (s, 9H).

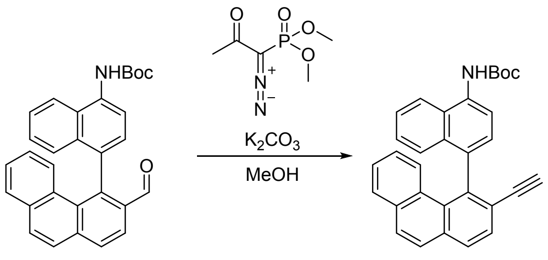

16 **17**

***tert*-Butyl (4-(3-ethynylphenanthren-4-yl)naphthalen-1-yl)carbamate (17).** To a 250 mL three-neck round bottom flask, **16** (1.727 g, 3.86 mmol, 1.0 eq), MeOH (77 mL), potassium carbonate (1.192 g, 8.63 mmol, 2.24 eq) and dimethyl (1-diazo-2-oxopropyl)phosphonate (1.0 mL, 5.4 mmol, 1.4 eq) were added. The reaction mixture was stirred at rt and monitored by LCMS. After overnight stirring, full consumption of **16** was indicated by LCMS, and the reaction mixture was transferred to a 1L separatory funnel, diluted with heptanes

(95 mL), EtOAc (180 mL), water (180 mL) and sat $NaHCO_3$ (95 mL). After separating layers, the aqueous layer was extracted with EtOAc (2 x 100 mL). The combined organic layers were washed with brine (100 mL), dried over Na_2SO_4 and concentrated *in vacuo* to yield a brown solid crude. The crude was dissolved in EtOAc and coated onto celite, then purified by column chromatography (1:9 EtOAc/heptanes). Fractions were checked with TLC (1:4 EtOAc/heptanes, UV and $KMnO_4$ staining), and fractions with R_f ~ 0.3 were combined and concentrated *in vacuo* to yield **17** as an off-white solid (1.256 g, 73% yield).

^{1}H NMR (400 MHz, $CDCl_3$) δ 8.05 (d, *J* = 7.7 Hz, 1H), 7.91 (d, *J* = 8.6 Hz, 1H), 7.85 (d, *J* = 8.2 Hz, 1H), 7.79 – 7.71 (m, 1H), 7.69 (dd, *J* = 9.5, 2.4 Hz, 3H), 7.41 (ddd, *J* = 8.3, 6.7, 1.3 Hz, 1H), 7.36 (dd, *J* = 10.5, 8.2 Hz, 2H), 7.32 (d, *J* = 8.5 Hz, 1H), 7.24 (t, *J* = 7.4 Hz, 1H), 7.16 (ddd, *J* = 8.2, 5.2, 1.1 Hz, 1H), 6.98 (s, 1H), 6.80 (ddd, *J* = 8.6, 6.9, 1.6 Hz, 1H), 2.65 (s, 1H), 1.53 (s, 9H).

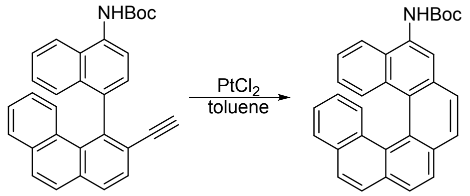

17 **2**

P/M-*tert*-Butyl hexahelicen-1-ylcarbamate (2). To a 500 mL three-neck round bottom flask, **17** (3.90 g, 8.79 mmol, 1.0 eq), platinum (II) chloride (240 mg, 0.902 mmol, 0.10 eq) and toluene (310 mL) were added. The reaction was heated to reflux for 21h, upon which full consumption of **17** was observed by LCMS. The reaction mixture was concentrated *in vacuo*, then dissolved in DCM, and an approximate same amount of heptanes was added. The resulting solution was concentrated at 700 mbar (50 °C water bath) until distillation stoped, then loaded onto a silica column for column chromatography (1:9 EtOAc/heptanes). Fractions were checked with TLC (1:4 EtOAc/heptanes), and fractions with R_f ~ 0.3 were combined and concentrated *in vacuo* to yield a pale yellow solid as desired product (2.45 g, 63% yield).

A sample of 150 mg was taken up in 75 mL EtOH:IPA (1:1) and the enantiomers were separated. The M enantiomer eluted first (77 mg, 99.2% *ee*). The P enantiomer eluted second (71 mg, 96.3% *ee)*.

^{1}H NMR (400 MHz, $CDCl_3$) δ 8.48 (s, 1H), 7.99 (d, *J* = 8.0 Hz, 2H), 7.96 (d, *J* = 7.2 Hz, 2H), 7.94 – 7.88 (m, 2H), 7.82 (ddd, *J* = 11.7, 8.1, 1.3 Hz, 2H), 7.69 (dd, *J* = 8.6, 1.2 Hz, 1H), 7.58 (dd, *J* = 8.5, 1.0 Hz, 1H), 7.26 (s, 1H), 7.20 (ddd, *J* = 8.0, 6.9, 1.2 Hz, 1H), 7.11 (s, 1H), 6.68 (dddd, *J* = 15.6, 8.4, 6.8, 1.3 Hz, 2H), 1.65 (s, 10H). Peak at 1.65 ppm shows 10H due to cumulative relaxation time difference.

Method for chiral preparative purification:

METHOD DESCRIPTION

**Prep SFC Method**	**:**	**100ml 30% A**
**Date**	**:**	**8 January 2025**
System	:	Sepiatec 250 SFC prep system with UV detector
Sample Name	:	MLN23000003-64
Sample Solvent	:	Ethanol/Isopropyl alcohol 1:1
Sample Concentration	:	2 mg/mL
Injection Volume	:	2000 µL
Column	:	Lux Cellulose-1 (250 x 21.20 mm; 5 µm) PN: 00G-4459-P0-AX
Column Temperature	:	42 °C
Total Flow	:	100 mL/Min
Back Pressure	:	120 Bar
Mobile Phase A	:	CO_2
Mobile Phase B	:	Methanol
Total Run Time	:	8.5 Min
UV Detection	:	264 nm
Pump Program	:	Isocratic at 30% mobile phase B

Analytical method for enantiomers:

METHOD DESCRIPTION

**Chiral SFC Method**	**:**	**23000003A UPC^2 6m Cel1 M**
System	:	Waters Acquity UPC^2 system with UV detector and QDA detector
Column	:	Acquity UPC^2 Trefoil CEL1 (3.0 x 150 mm; 2.5 µm)
Mobile Phase A	:	CO_2
Mobile Phase B	:	Methanol
Pump Flow	:	1.5 mL/Min
UV Detection	:	264 nm
Injection Volume	:	1.0 µL
Total Run Time	:	6.0 Min
Column Temperature	:	40 °C
ABPR	:	2000 psi
Mass Detection	:	MS Scan ES positive and negative
Mass Range	:	100 – 1250 Da
Pump Program	:	Gradient

Time (Min)	%A	%B	Curve
Initial	98.0	2.0	Initial
3.5	60.0	40.0	6
5.0	60.0	40.0	6
5.1	98.0	2.0	11

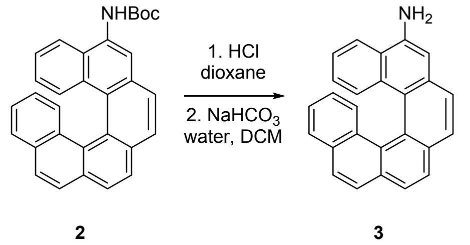

P-Hexahelicen-1-amine (3). Compound P-**2** (54.0 mg, 122 µmol, 1.0 eq.) was stirred in HCl in dioxane (4 M, 600 µL, HCl in excess.) at room temperature overnight. The reaction mixture was filtered and the pale-brown solid was washed with cold Et_2O (5 mL) to give P-**3**·HCl (31.8 mg). The material was characterized via ^{1}HNMR in CD_3OD, and once its structure was confirmed, P-**3**·HCl (25.3 mg) was partitioned between DCM (5 mL) and $NaHCO_3$ (aq., satd., 2.5 mL). The aqueous phase was extracted with DCM (5 mL). The combined organic phases were dried over Na_2SO_4, filtered, and concentrated *in vacuo* to give P-**3** (21.6 mg) as a pale-yellow solid, which was characterized via ^{1}HNMR in DMSO-d6).

^{1}H NMR P-**3**·HCl (400 MHz, MeOD) δ 8.18 (t, J = 8.2 Hz, 0H), 8.12 – 8.06 (m, 0H), 8.05 – 7.96 (m, 0H), 7.89 (dd, J = 8.1, 1.4 Hz,0H), 7.66 (d, J = 8.6 Hz, 0H), 7.52 – 7.37 (m, 0H), 7.23 (ddd, J = 8.0, 6.9, 1.1 Hz, 0H), 6.82 (ddd, J = 8.4, 6.9, 1.3 Hz, 0H), 6.61 (ddd, J = 8.4, 6.9, 1.4 Hz, 0H), 4.89 (s, 1H)

^{1}H NMR (299 MHz, DMSO-d_6) δ 8.12 (d, *J* = 8.3 Hz, 1H), 8.07 – 7.92 (m, 5H), 7.89 (d, *J* = 7.9 Hz, 1H), 7.82 (d, *J* = 8.3 Hz, 1H), 7.54 – 7.42 (m, 2H), 7.22 (t, *J* = 7.5 Hz, 2H), 7.08 (s, 1H), 6.70 – 6.58 (m, 2H), 6.10 (br, 2H).

Synthesis towards helicenes 1, 4 and 5

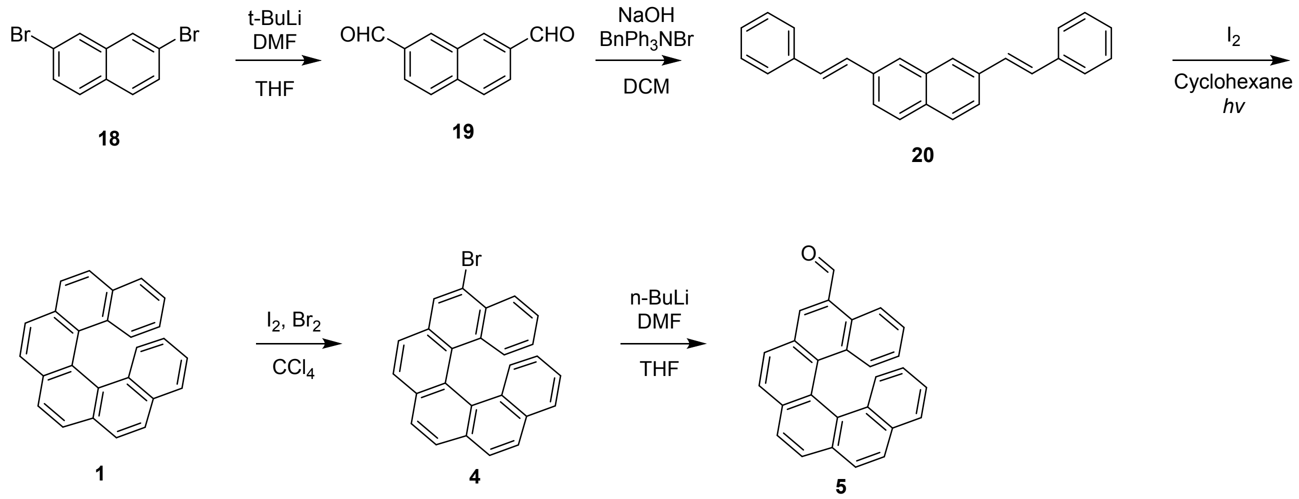

Scheme 4: Synthesis towards helicenes **1**, **4** and **5**.

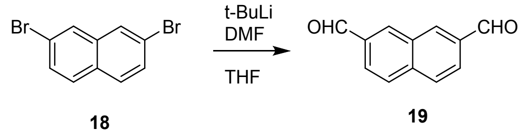

Naphthalene-2,7-dicarbaldehyde (19). In a RBF, 2,7-dibromonaphthalene (**18**, 1.00 g, 1 Eq, 3.50 mmol) was dissolved in dry THF (35 mL) under nitrogen. The reaction mixture was cooled to T < -78 °C. *t*-BuLi (1.16 g, 9.57 mL, 1.90 molar, 5.20 Eq, 18.2 mmol) was added dropwise in 15 minutes (T < -78 °C). The reaction mixture was stirred for 1 h (T < -78 °C). DMF (1.27 g, 1.34 mL, 4.95 Eq, 17.3 mmol) in THF (~ 2 mL) was added dropwise in 10 minutes (T < -78 °C). 12.30 The reaction mixture was stirred for 30 minutes (T < -78 °C). The

reaction mixture was allowed to reach -30 °C (50 minutes). ^{1}H NMR showed relatively pure product. HCl (12%, aq., 3.0 mL) and water (20 mL) were mixed and added dropwise. The layers were separated. The aqueous phase was extracted with DCM (2 × 20 mL). The combined organic phases were dried over Na_2SO_4, filtered, and concentrated *in vacuo* to give a yellow solid (643 mg). Automated NP-column chromatography (silica gel (25 g), gradient EtOAc : heptanes = 0 : 1 → 2 : 1, UV, excessive tailing) was performed. Pure fractions by TLC (silica gel, EtOAc : heptanes = 1 : 3, UV) were pooled. Concentration *in vacuo* gave **19** (602 mg, 3.27 mmol, 93.4%) as a white solid. LCMS showed a purity of 99% (at 210 - 500 nm) and 97% (at 254 nm) and a m/z of 157.4 ((M-CHO+H)$^+$).

^{1}H NMR (299 MHz, $CDCl_3$) δ 10.24 (s, 2H), 8.58 – 8.48 (m, 2H), 8.15 (dd, *J* = 8.6, 1.5 Hz, 2H), 8.05 (d, *J* = 8.5 Hz, 2H).

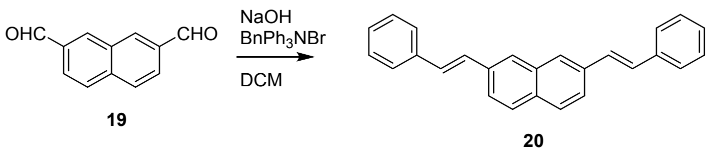

2,7-distyrylnaphthalene (20). In a RBF, compound **19** (500 mg, 1 Eq, 2.71 mmol) and benzyltriphenylphosphanium bromide (2.35 g, 2.0 Eq, 5.43 mmol) were suspended in DCM (50 mL) under nitrogen atmosphere. To the mixture, sodium hydroxide aq (4.34 g, 50% Wt, 20 Eq, 54.3 mmol) was added dropwise keeping the temperature below 28°C. The beige suspension was stirred at r.t. overnight. LCMS showed complete conversion towards desired compound and water (~ 50 mL) was added to the reaction mixture. The aqueous phase was extracted with DCM (3 × 50 mL). The combined organic phases were dried over Na_2SO_4, filtered over small pad of silicagel, and concentrated *in vacuo* to give a yellow solid. Automated NP-column chromatography (dry coating on 5 g silica gel, column silica gel (25 g); gradient DCM: heptanes = 0 : 1 → 2: 4, UV) was performed. Concentration *in vacuo* gave **20** (568 mg, 1.71 mmol, 62.9%) as a white solid (mixture of isomers). LCMS showed a purity of 97% (at 210 - 500 nm)

^{1}H NMR (400 MHz, $CDCl_3$) δ 7.80 – 7.67 (m, 3H), 7.67 – 7.51 (m, 5H), 7.44 – 7.35 (m, 2H), 7.35 – 7.25 (m, 6H), 7.25 – 7.13 (m, 5H), 6.80 – 6.64 (m, 4H).

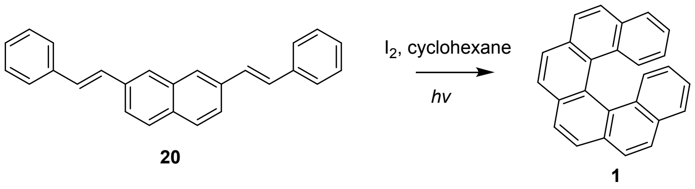

P/M-Hexahelicene (1). Compound **20** (400 mg, 1 Eq, 1.20 mmol, mixture of isomers) and I_2 (678 mg, 2.22 Eq, 2.67 mmol) were dissolved in cyclohexane (800 mL). 1,2-Epoxy-isobutane (2.65 g, 3.28 mL, 30.55 Eq, 36.8 mmol) was added and the reaction started. The purple reaction mixture was irradiated with a Philips PL-L 36W 01, UV-B lamp using a flow system (20 mL/min) with water cooling where the irradiated reaction mixture was pumped back in the flask containing the stock solution. The reaction progress was followed by HPLC, complete conversion was achieved in 72h. The reaction mixture was concentrated to dryness then 100 mL chloroform was added. The organic washed with $Na_2S_2O_3$ (aq., 1 M, 2 × 50 mL) until absence of the purple colour. The water layer was extracted with 50 mL chloroform and the combined organic phases were dried over Na_2SO_4, filtered, and concentrated *in vacuo* to give a brown solid (670 mg). Automated NP-column chromatography (dry coating on hydromatrix, silica gel (25 g); gradient DCM: heptanes 0:1 → 2:3) was performed. Concentration *in vacuo* afforded **1** as a yellow solid (308 mg, 938 µmol, 77.9%).
A sample of 50 mg racemate was taken up in MeCN (7 mL) and the enantiomers were separated using a prep SFC system to furnish 9 mg of the first eluting peak (99% *ee*) which corresponded to the M enantiomer.

H NMR (400 MHz, $CDCl_3$) δ 8.04 – 7.97 (m, 4H), 7.96 – 7.90 (m, 4H), 7.83 (ddd, *J* = 8.2, 1.6, 0.6 Hz, 2H), 7.59 (ddd, *J* = 8.6, 1.2, 0.6 Hz, 2H), 7.22 (ddd, *J* = 8.0, 6.9, 1.1 Hz, 2H), 6.68 (ddd, *J* = 8.4, 6.9, 1.4 Hz, 2H).

Preparative SFC conditions:

METHOD DESCRIPTION

Prep SFC Method : **100ml 25% B**
Date : **2 October 2024**

System : Sepiatec 250 SFC prep system with UV detector
Sample Name : BBN23000003-99
Sample Solvent : Acetonitrile
Sample Concentration : 6 mg/mL
Injection Volume : 500 µL
Column : Lux Cellulose-1 (250 x 21.20 mm; 5 µm) PN: 00G-4459-P0-AX
Column Temperature : 42 °C
Total Flow : 100 mL/Min
Back Pressure : 120 Bar
Mobile Phase A : CO_2
Mobile Phase B : Methanol + 0.2% NH_4OH (25%, aq)
Total Run Time : 11.5 Min
UV Detection : 252 nm
Pump Program : Isocratic at 25% mobile phase B

Analytical method for enantiomers:

METHOD DESCRIPTION

Chiral SFC Method : **23000003B UPC^2 6m Cel3 MA1**

System : Waters Acquity UPC^2 system with UV detector and QDA detector
Column : Phenomenex Lux Cellulose 3 (3.0 x 150 mm; 3 µm)
Mobile Phase A : CO_2
Mobile Phase B : Methanol + 0.2% NH_4OH (25%, aq)
Pump Flow : 2.0 mL/Min
UV Detection : 252 nm
Injection Volume : 2.0 µL
Total Run Time : 6.0 Min
Column Temperature : 40 °C
ABPR : 2000 psi
Mass Detection : MS Scan ES positive and negative
Mass Range : 100 – 600 Da
Pump Program : Gradient

Time (Min)	%A	%B	Curve
Initial	98.0	2.0	Initial
5.0	65.0	35.0	6
5.1	98.0	2.0	6

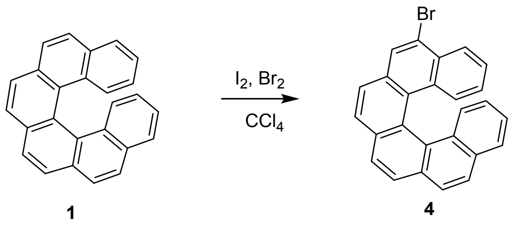

P/M-1-Bromohexahelicene (4). In a RBF hexahelicene (150 mg, 1 Eq, 457 µmol) and iodine (5.80 mg, 0.05 Eq, 22.8 µmol) were dissolved in carbon tetrachloride (15 mL). A solution of bromine (73.0 mg, 23.5 µL, 1.00 Eq, 457 µmol) in carbon tetrachloride (2.0 mL) was added dropwise. The reaction was stirred overnight at room temperature in the dark. The RM was concentrated under vacuum to a brown oil with some solid. The residue was partially dissolved in DMSO, filtered and purified by reversed phase column chromatography in two portions. The fractions containing product were pooled and concentrated to dryness affording 33 mg of racemic **4**.

Racemic **4** was dissolved in DCM-cyclohexane (total volume approx. 5 mL, mainly DCM) and then purified by chiral prep chromatography to furnish 10 mg of yellow solid 99.6% ee and 99% purity (first eluting peak, P enantiomer) and 15 mg of a yellow oil-solid of 84.2% ee (second eluting peak, M enantiomer).

^{1}H NMR (400 MHz, $CDCl_3$) δ 8.27 (d, *J* = 7.3 Hz, 2H), 8.07 – 7.98 (m, 4H), 7.93 (s, 1H), 7.89 (d, *J* = 8.2 Hz, 1H), 7.82 (ddd, *J* = 8.0, 1.4, 0.6 Hz, 1H), 7.68 – 7.59 (m, 1H), 7.59 – 7.48 (m, 1H), 7.31 (ddd, *J* = 8.2, 6.9, 1.2 Hz, 1H), 7.22 (ddd, *J* = 8.0, 6.9, 1.2 Hz, 2H), 6.71 (dddd, *J* = 8.3, 6.8, 5.2, 1.4 Hz, 2H).

Method for chiral preparative purification:

METHOD DESCRIPTION

**Prep LC Method**	:	**23000003-106-prep**
**Date**	:	**01-Oct-2024**
System	:	Agilent 1200 series with UV detector
Sample Name	:	BBN23000003-106
Sample Solvent	:	DCM/Cyclopentane (2/1)
Sample Concentration	:	8 mg/mL
Injection Volume	:	50 µL
Column	:	Chiralpak IG (250 x 21 mm; 5 µm)
Column Temperature	:	Ambient
Total Flow	:	20 mL/Min
Mobile Phase A	:	Heptane/DCM/Ethanol (85/15/0.5)
Total Run Time	:	6 Min
UV Detection	:	215 + 254 nm
Pump Program	:	Isocratic

Analytical method for enantiomers:

METHOD DESCRIPTION

**Chiral SFC Method**	:	**23000003D UPC^2 12m IG MA1**
System	:	Waters Acquity UPC^2 system with UV detector and QDA detector
Column	:	Daicel Chiralpak IG-3 (3.0 x 150 mm; 3 µm)
Mobile Phase A	:	CO_2
Mobile Phase B	:	Methanol + 0.2% NH_4OH (25%, aq)
Pump Flow	:	1.0 mL/Min
UV Detection	:	254 nm
Injection Volume	:	2.0 µL
Total Run Time	:	12.0 Min
Column Temperature	:	40 °C
ABPR	:	2000 psi
Mass Detection	:	MS Scan ES positive and negative
Mass Range	:	100 – 1250 Da
Pump Program	:	Gradient

Time (Min)	%A	%B	Curve
Initial	98.0	2.0	Initial
6.0	60.0	40.0	6
9.0	60.0	40.0	6
11.1	98.0	2.0	6

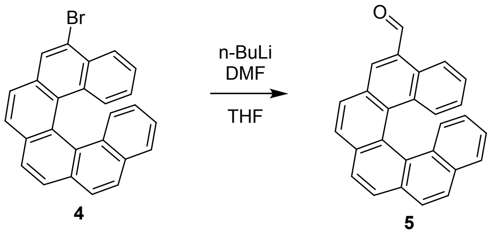

4 5

P/M-hexahelicene-1-carbaldehyde (5). Compound P-**4** (40 mg, 1 Eq, 98 µmol) was dissolved in dry THF (6.00 mL). The solution was cooled to < -78 °C (dry ice-acetone bath). n-BuLi (16 mg, 98 µL, 2.50 molar, 2.50 Eq, 0.25 mmol) was added dropwise (T < -78 °C). The yellow reaction was stirred at - 78 °C for 5 min. Dry DMF (0.14 g, 0.15 mL, 20 Eq, 2.0 mmol) was added dropwise (T < -78 °C). The reaction was continued at - 78 °C for 2 h. The reaction was quenched by the dropwise addition of NH_4Cl (aq., sat., 1 mL, T < -60 °C). The reaction was allowed to reach room temperature and was partitioned between water (10 mL) and DCM (10 mL). The water phase was extracted with DCM (2 × 10 mL). The combined organic phases were washed with water (10 mL) and brine (10 mL), dried over Na_2SO_4, filtered, and concentrated *in vacuo* to give a yellow solid. The material was submitted for prep HPLC by dissolving it in a mixture of DMSO: THF (1:3, 2 mL) to give P-**5** as a yellow solid (4 mg, 10%) with an enantiomeric purity of 97%.

^{1}H NMR (400 MHz, $CDCl_3$) δ 10.55 (s, 1H), 9.20 (ddd, *J* = 8.3, 1.3, 0.6 Hz, 1H), 8.49 (s, 1H), 8.10 (s, 2H), 8.09 – 8.01 (m, 2H), 7.95 (s, 2H), 7.83 (ddd, *J* = 8.0, 1.4, 0.6 Hz, 1H), 7.62 (dt, *J* = 8.5, 1.1 Hz, 1H), 7.50 – 7.45 (m, 1H), 7.34 (ddd, *J* = 8.3, 6.9, 1.3 Hz, 1H), 7.25 – 7.20 (m, 1H), 6.72 (dtd, *J* = 8.4, 6.9, 1.4 Hz, 2H).

Analytical method for enantiomers:

METHOD DESCRIPTION

Chiral SFC Method : 23000003F UPC² 10m Cel1 MA1

System	:	Waters Acquity UPC² system with UV detector and QDA detector
Column	:	Acquity UPC² Trefoil CEL1 (3.0 x 150 mm; 2.5 µm)
Mobile Phase A	:	CO_2
Mobile Phase B	:	Methanol + 0.2% NH_4OH (25%, aq)
Pump Flow	:	2.5 mL/Min
UV Detection	:	246 nm
Injection Volume	:	3.0 µL
Total Run Time	:	10.0 Min
Column Temperature	:	40 °C
ABPR	:	2000 psi
Mass Detection	:	MS Scan ES positive and negative
Mass Range	:	100 – 600 Da
Pump Program	:	Gradient

Time (Min)	%A	%B	Curve
Initial	98.0	2.0	Initial
6.0	60.0	40.0	6
9.0	60.0	40.0	6
9.1	98.0	2.0	6

Below, all the ^{1}H-NMR analyses are reported.

^{1}H NMR (400 MHz, $CDCl_3$) spectrum of *tert*-butyl (4-bromonaphthalen-1-yl)carbamate (**7**)

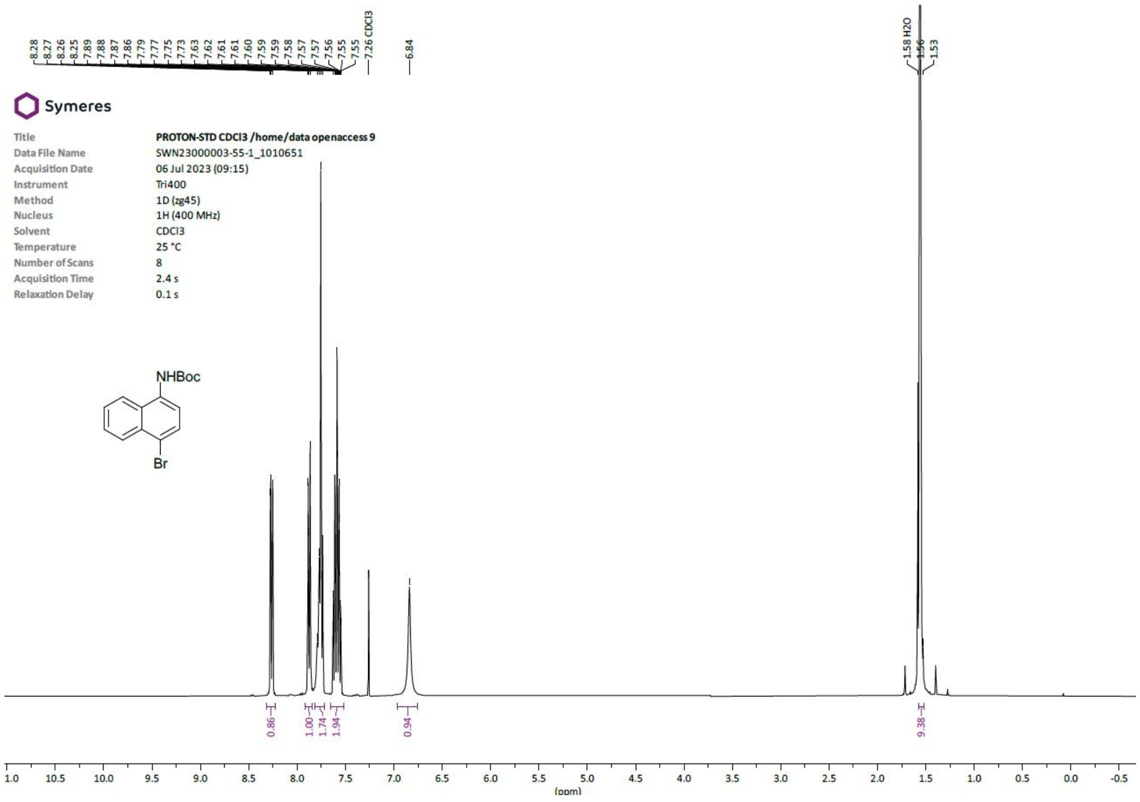

^{1}H NMR (400 MHz, DMSO-*d6*) spectrum of (4-((tert-butoxycarbonyl)amino)naphthalen-1-yl)boronic acid (**8**)

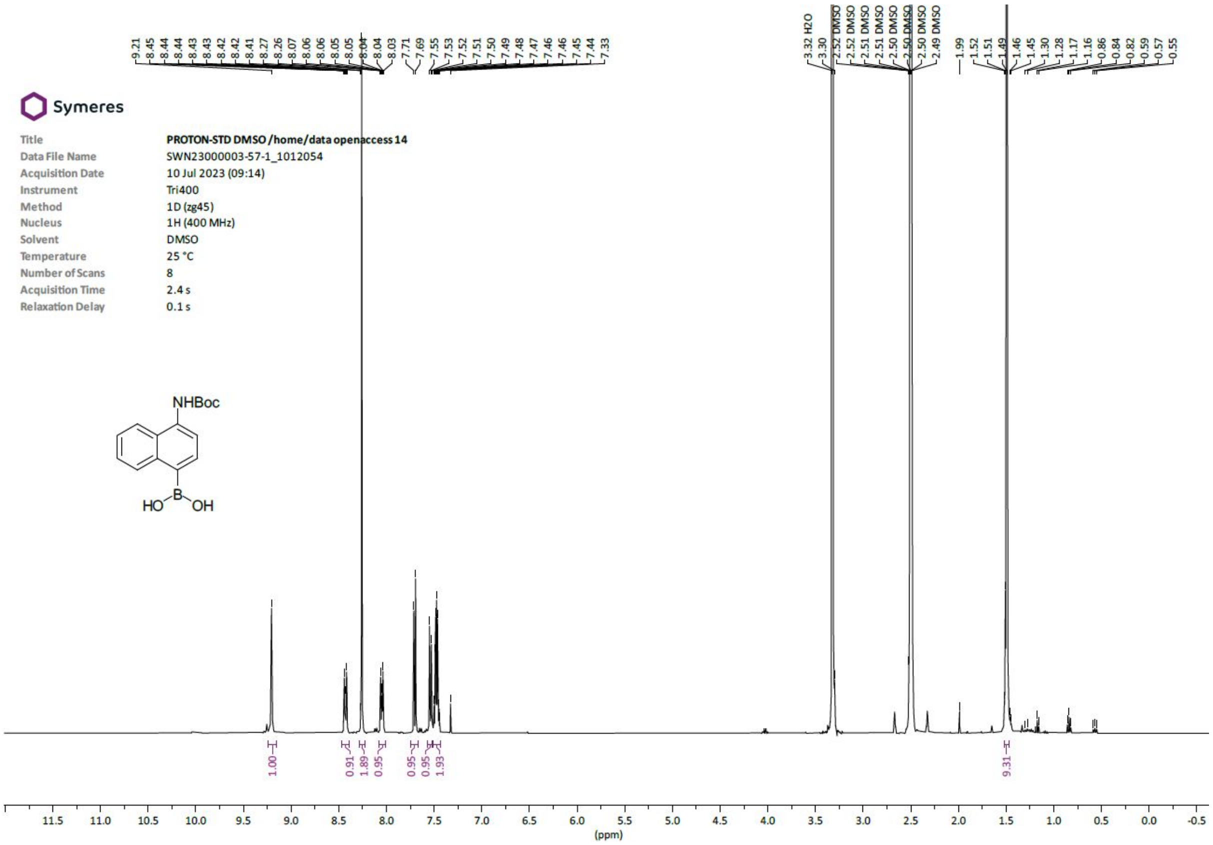

^{1}H NMR (400 MHz, DMSO-*d6*) spectrum of 4-(naphthalen-2-yl)-4-oxobutanoic acid (**11**)

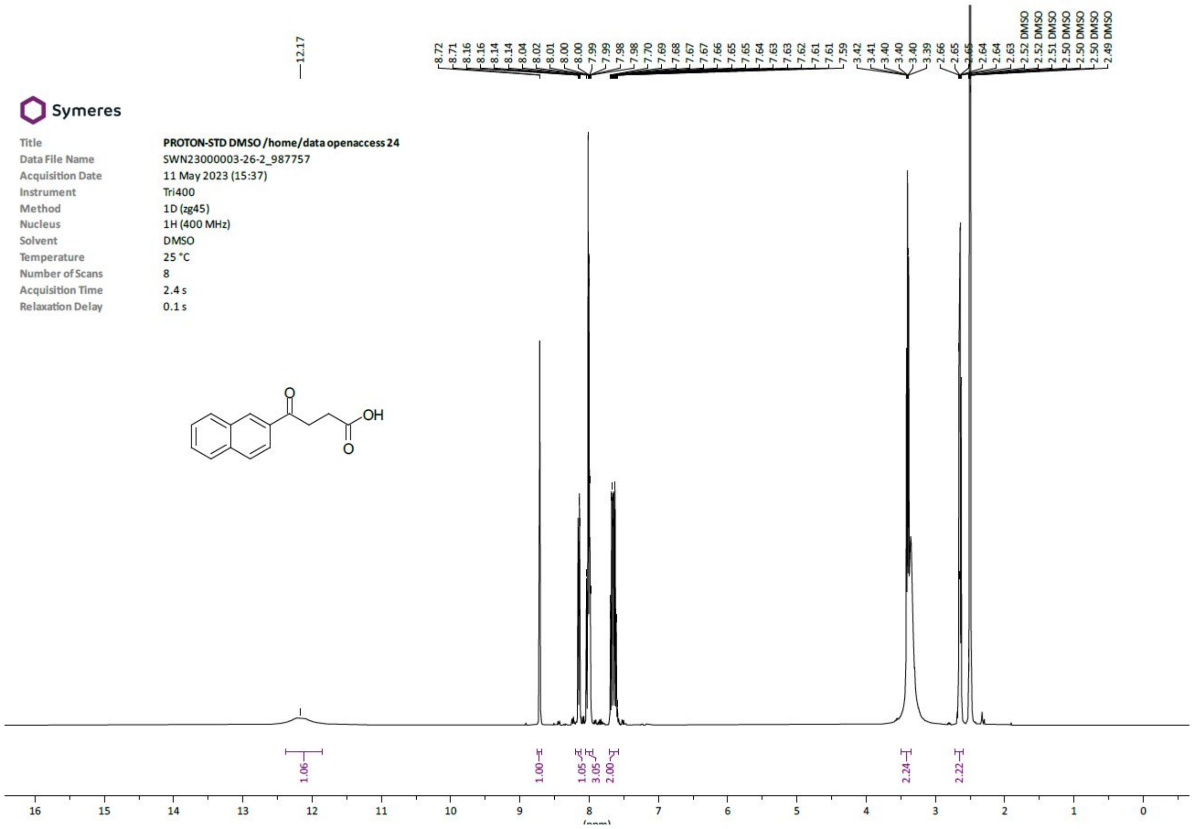

^1H NMR (400 MHz, $CDCl_3$) spectrum of 4-(naphthalen-2-yl)butanoic acid (**12**)

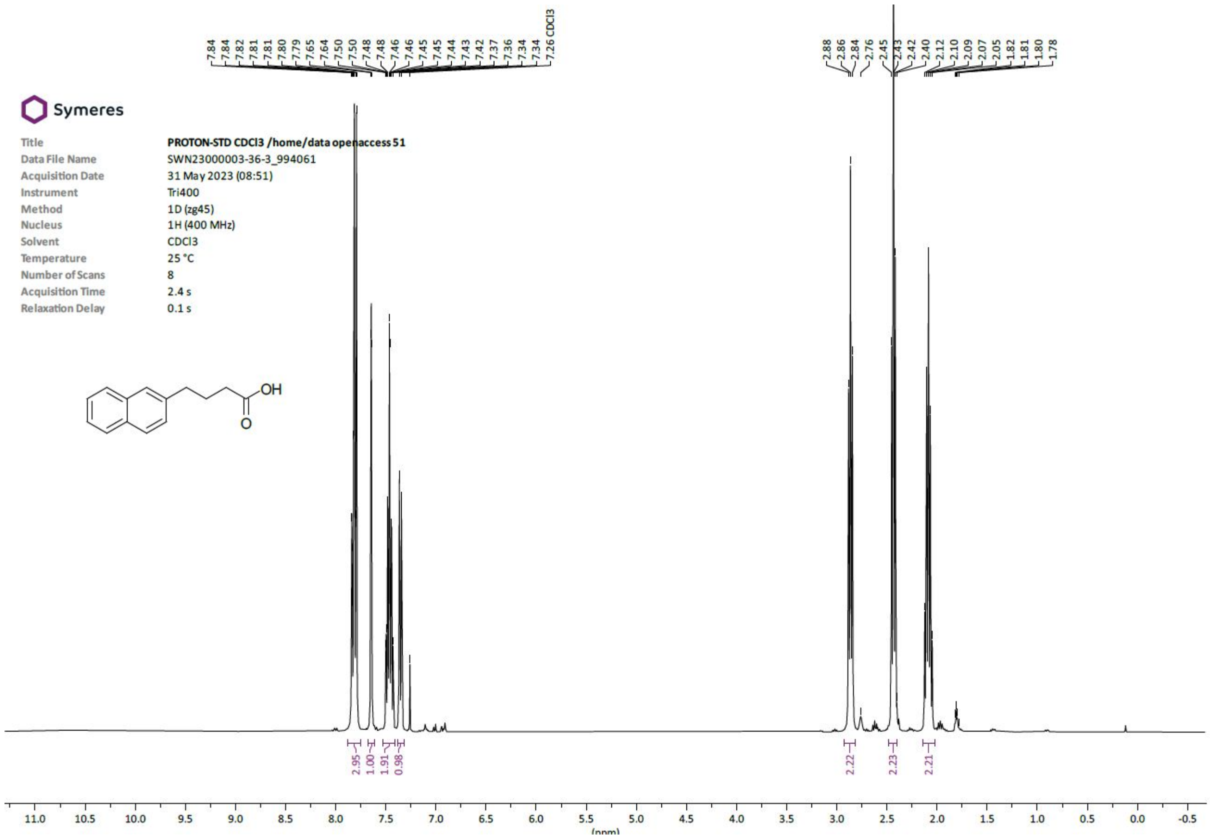

^1H NMR (400 MHz, $CDCl_3$) spectrum of 2,3-dihydrophenanthren-4(1H)-one (**13**)

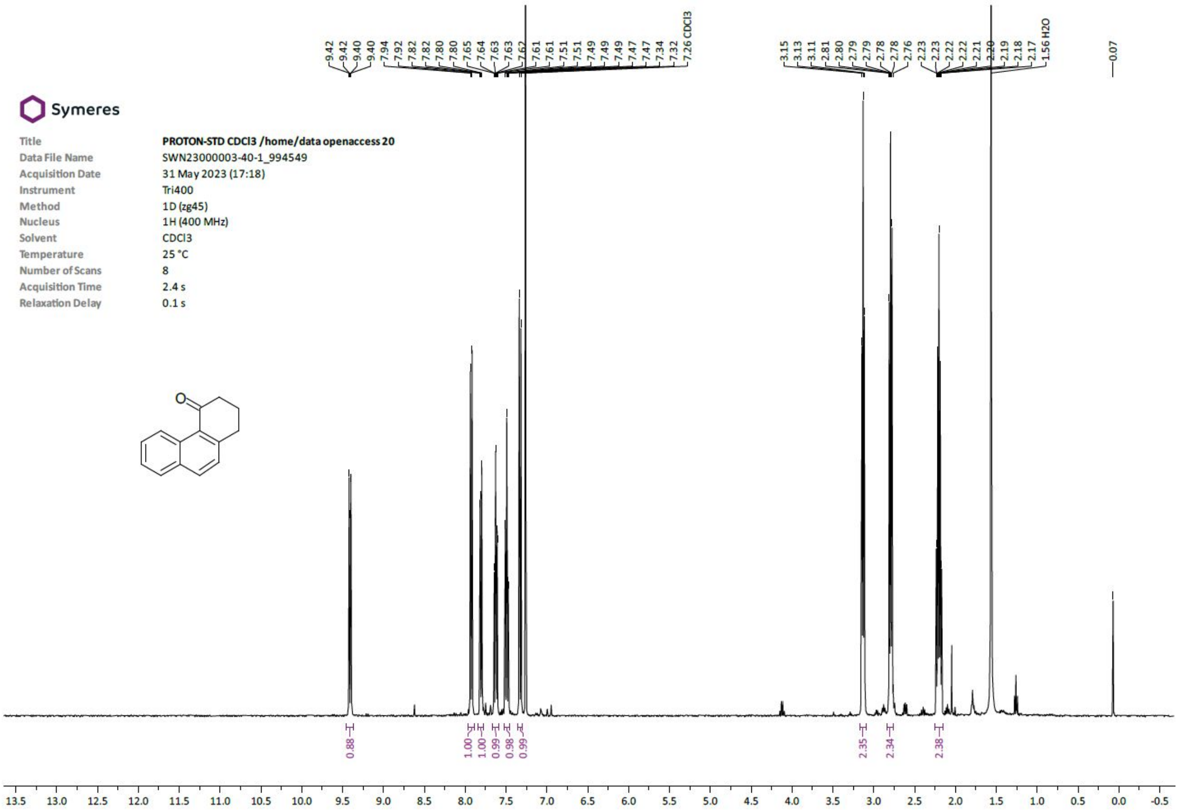

^1H NMR (400 MHz, $CDCl_3$) spectrum of 4-bromo-1,2-dihydrophenanthrene-3-carbaldehyde (**14**)

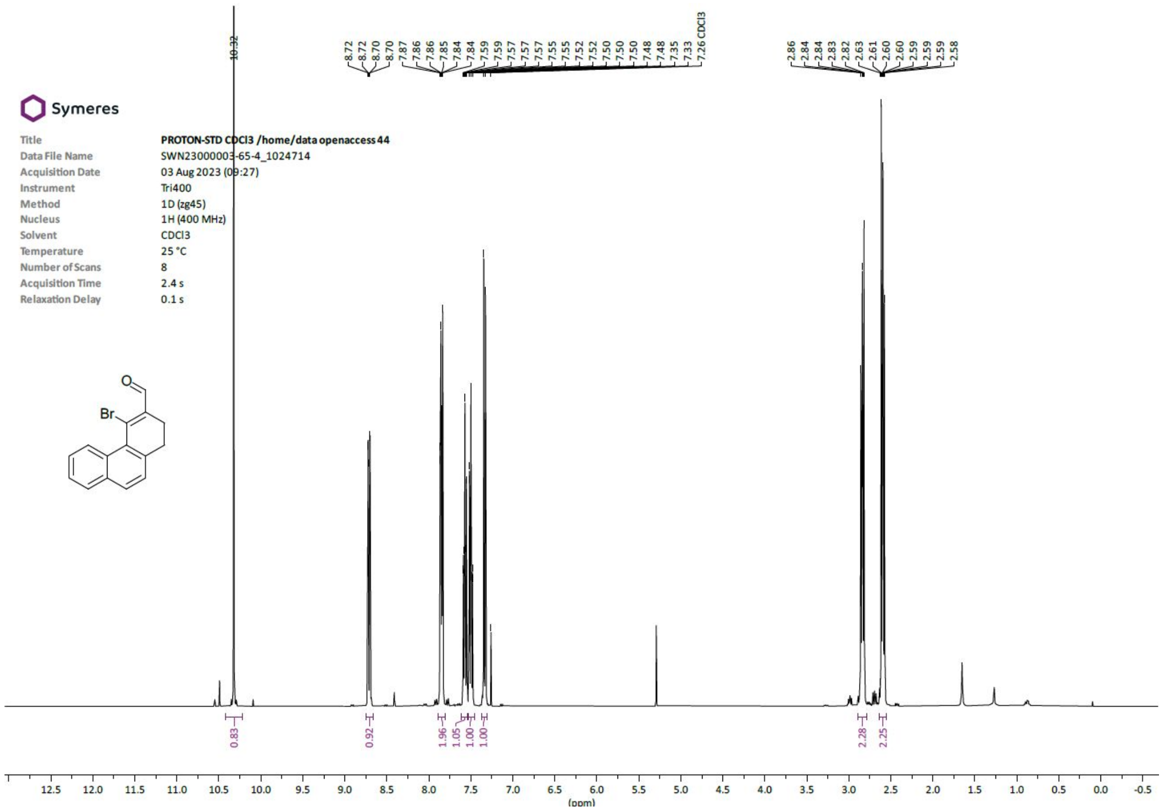

^{1}H NMR (400 MHz, $CDCl_3$) spectrum of *tert*-butyl (4-(3-formyl-1,2-dihydrophenanthren-4-yl)naphthalen-1-yl)carbamate (**15**)

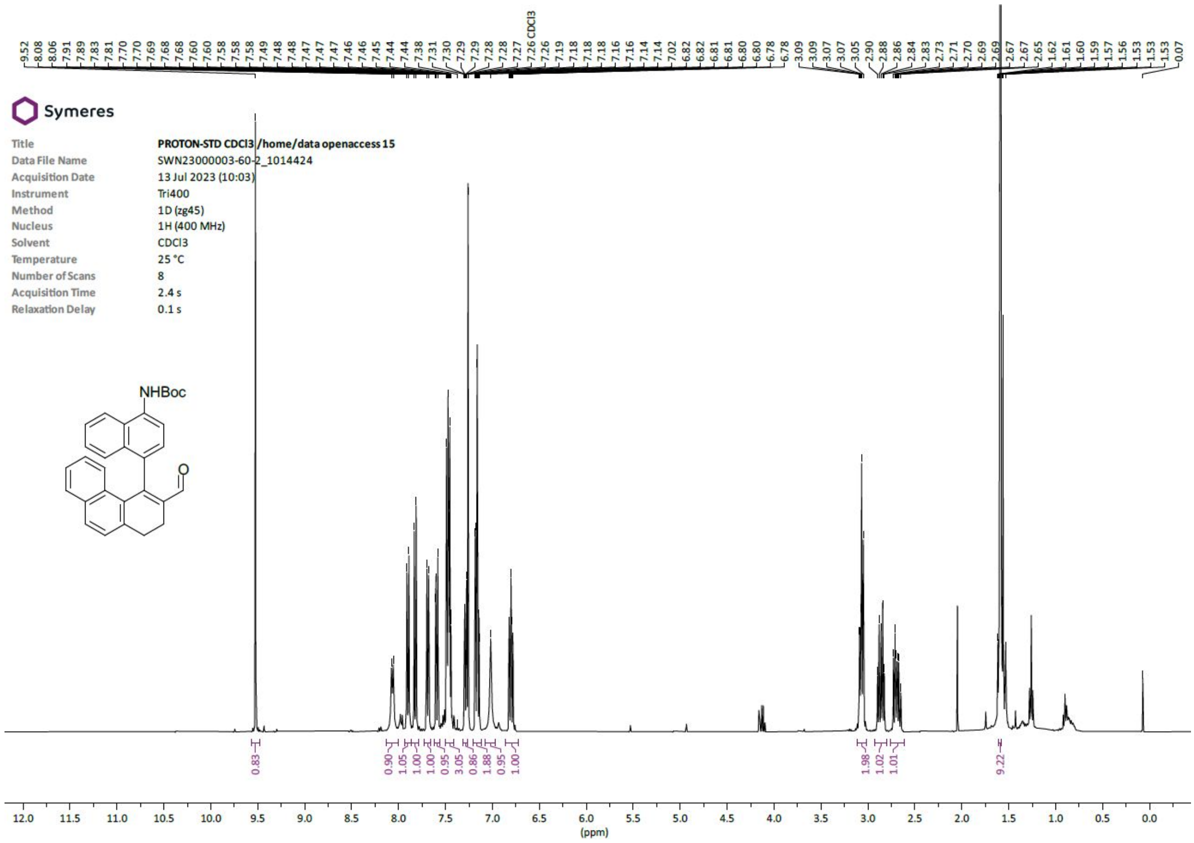

^{1}H NMR (400 MHz, $CDCl_3$) spectrum of *tert*-butyl (4-(3-formylphenanthren-4-yl)naphthalen-1-yl)carbamate (**16**)

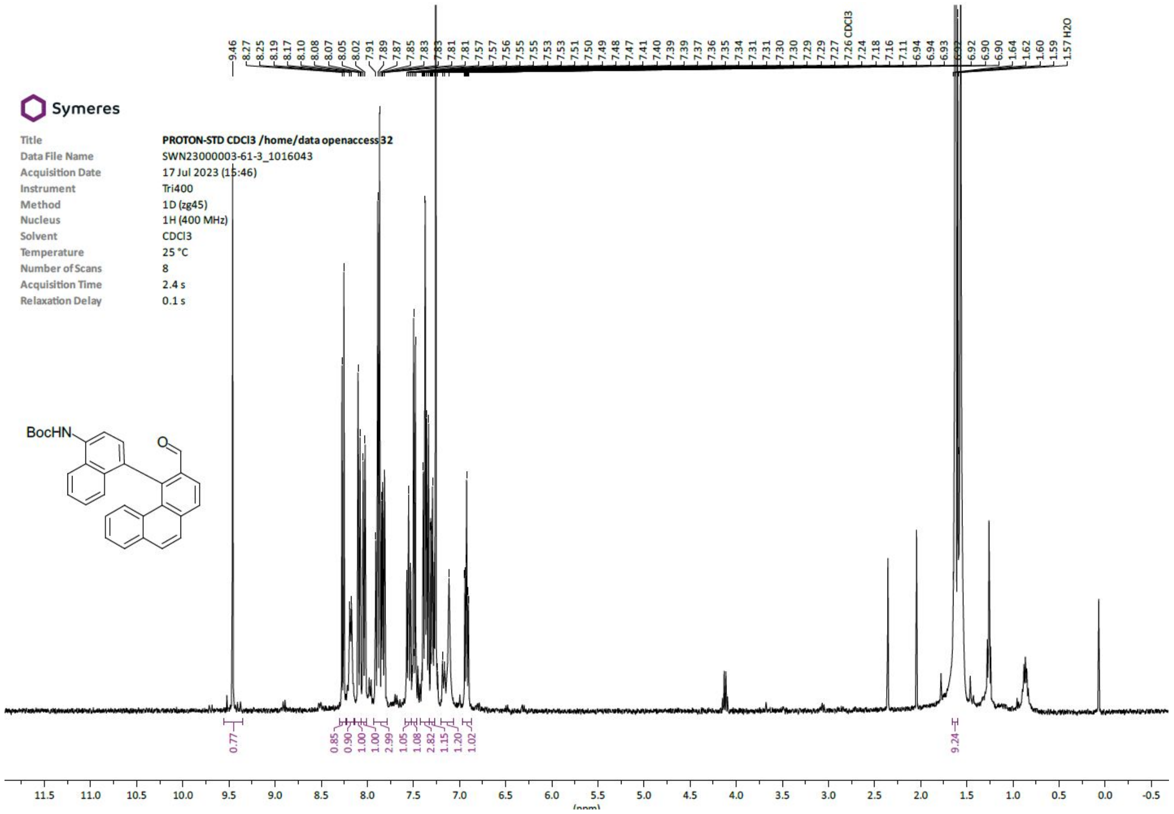

^{1}H NMR (400 MHz, $CDCl_3$) spectrum of *tert*-butyl (4-(3-ethynylphenanthren-4-yl)naphthalen-1-yl)carbamate (**17**)

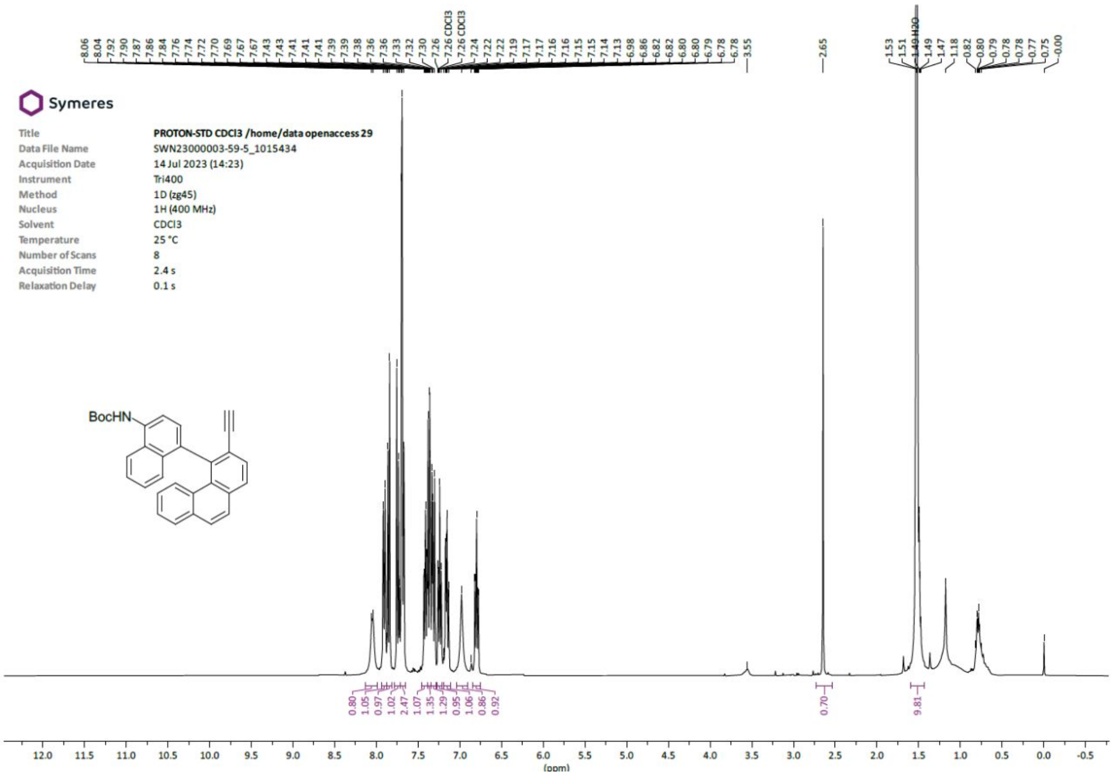

^{1}H NMR (400 MHz, $CDCl_3$) spectrum of *tert*-butyl hexahelicen-1-ylcarbamate (**2**)

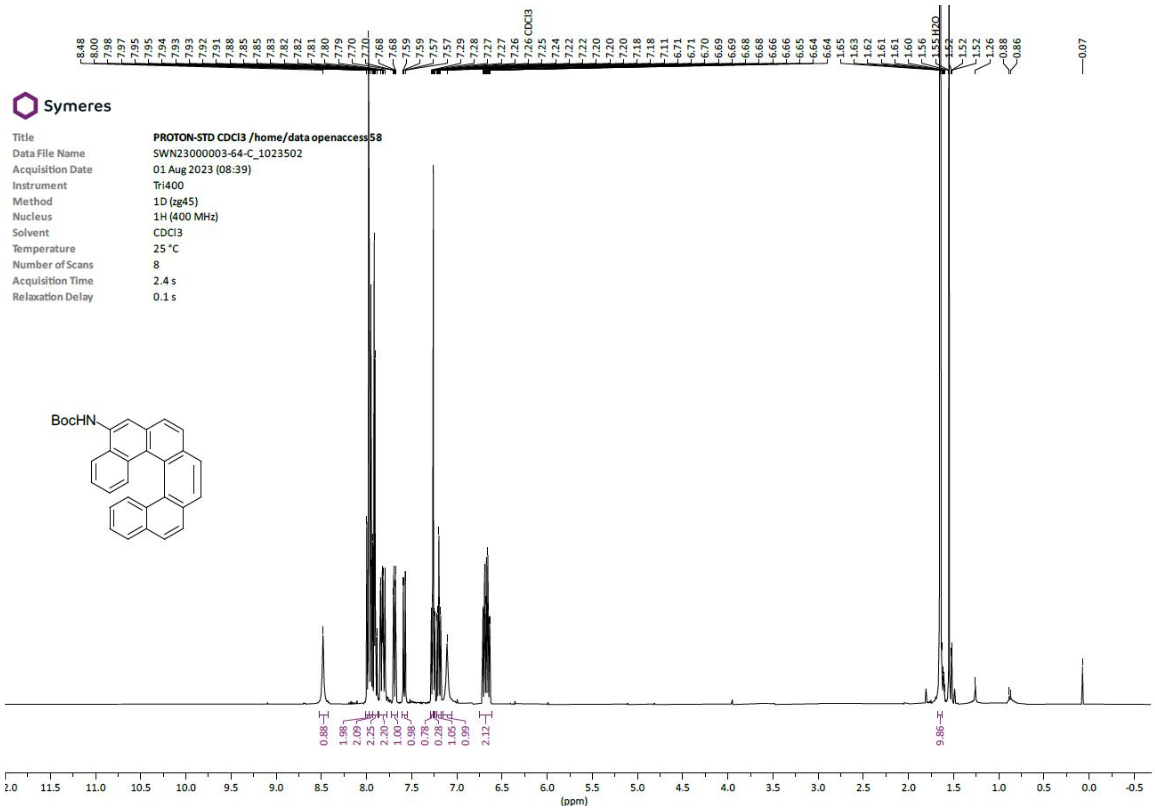

^{1}H NMR (400 MHz, MeOD) spectrum of P-hexahelicen-1-amine hydrochloride (P-**3***HCl, Crude)

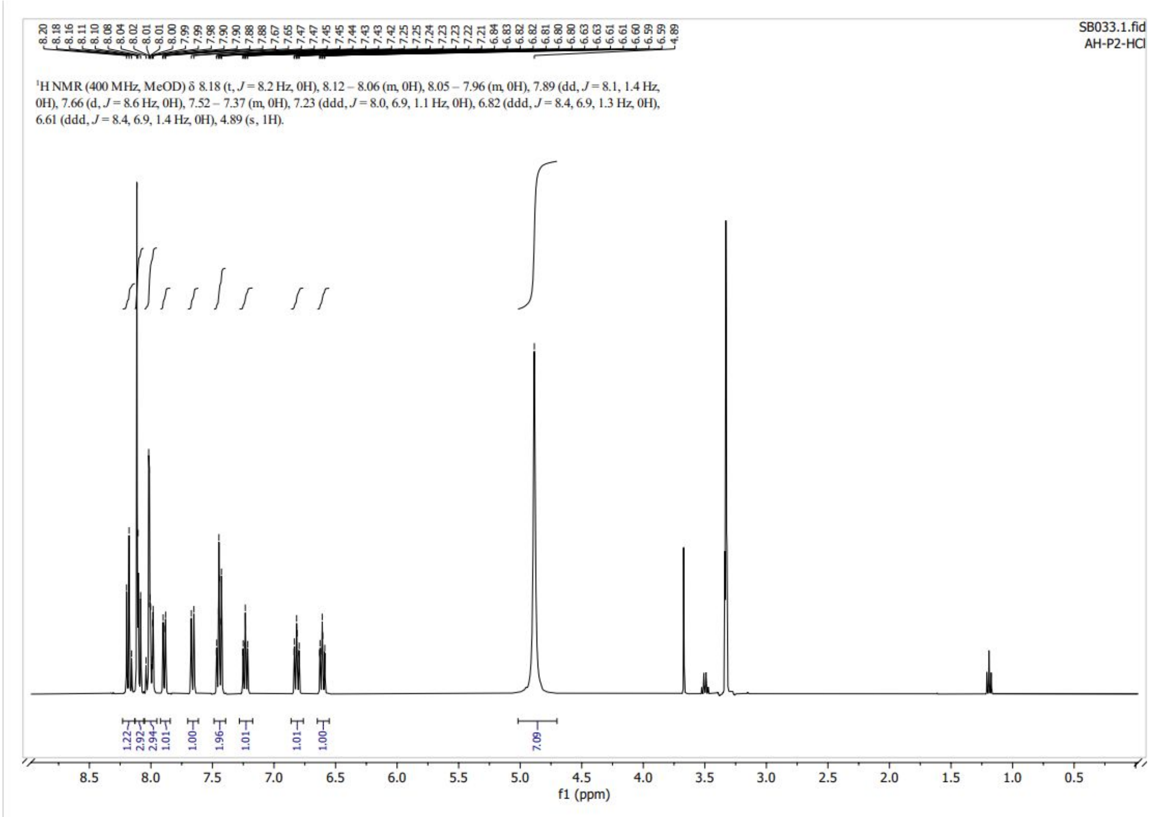

^{1}H NMR (400 MHz, DMSO-d6) spectrum of P-hexahelicen-1-amine (P-**3**, Crude)

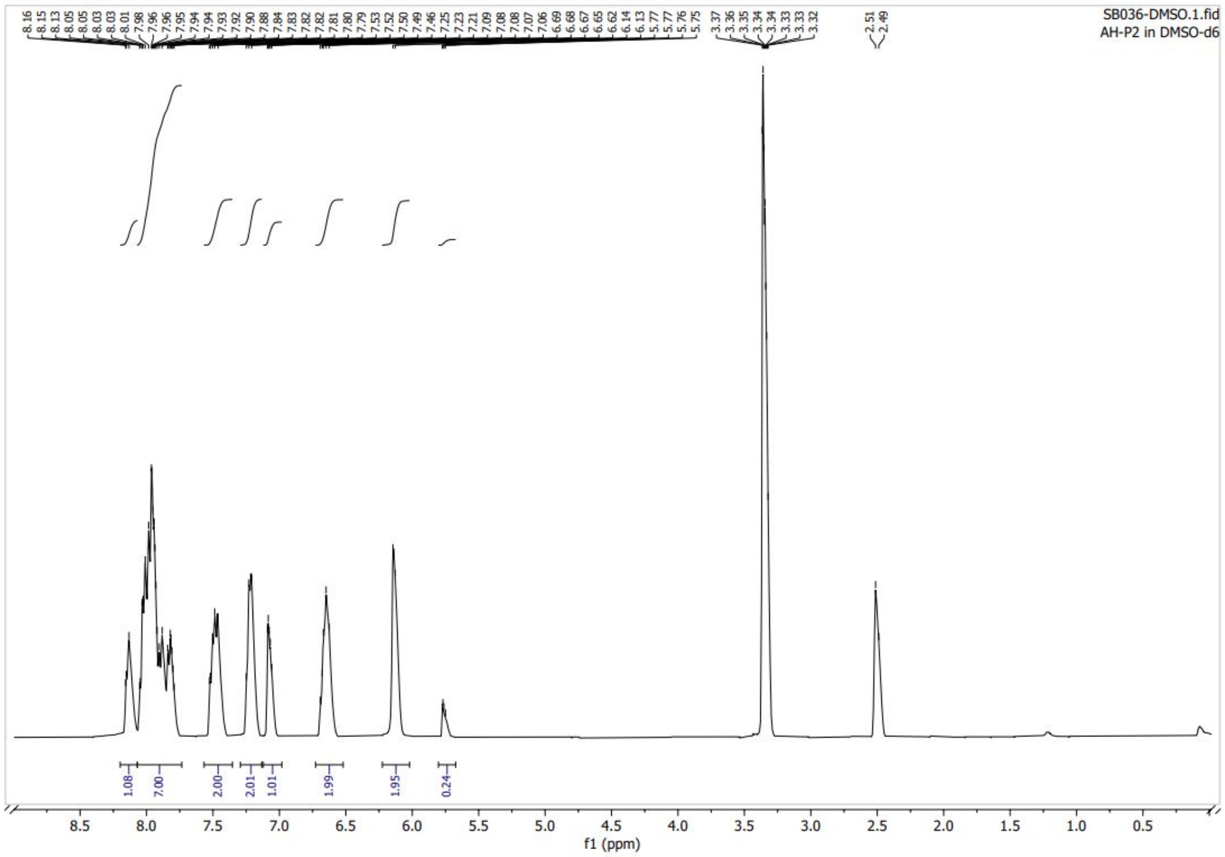

^{1}H NMR (400 MHz, $CDCl_3$) spectrum of Naphthalene-2,7-dicarbaldehyde (**19**)

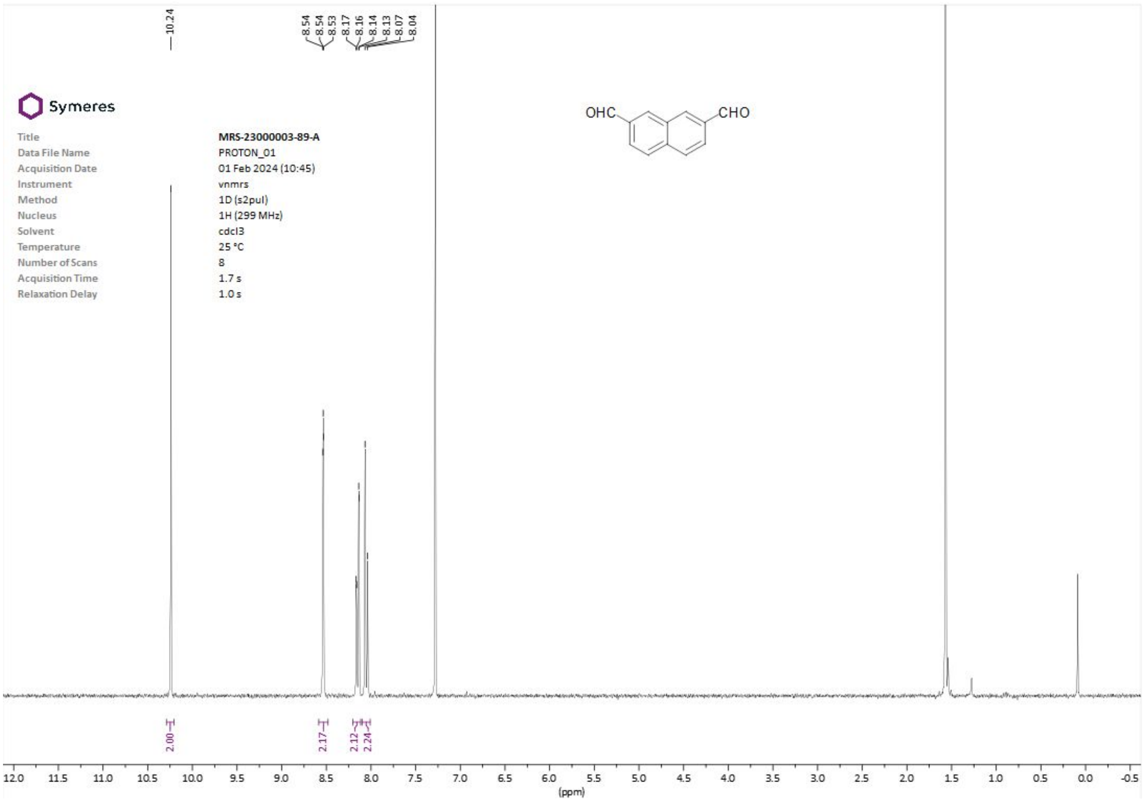

^{1}H NMR (400 MHz, $CDCl_3$) spectrum of 2,7-di((E)-styryl)naphthalene **(20)**

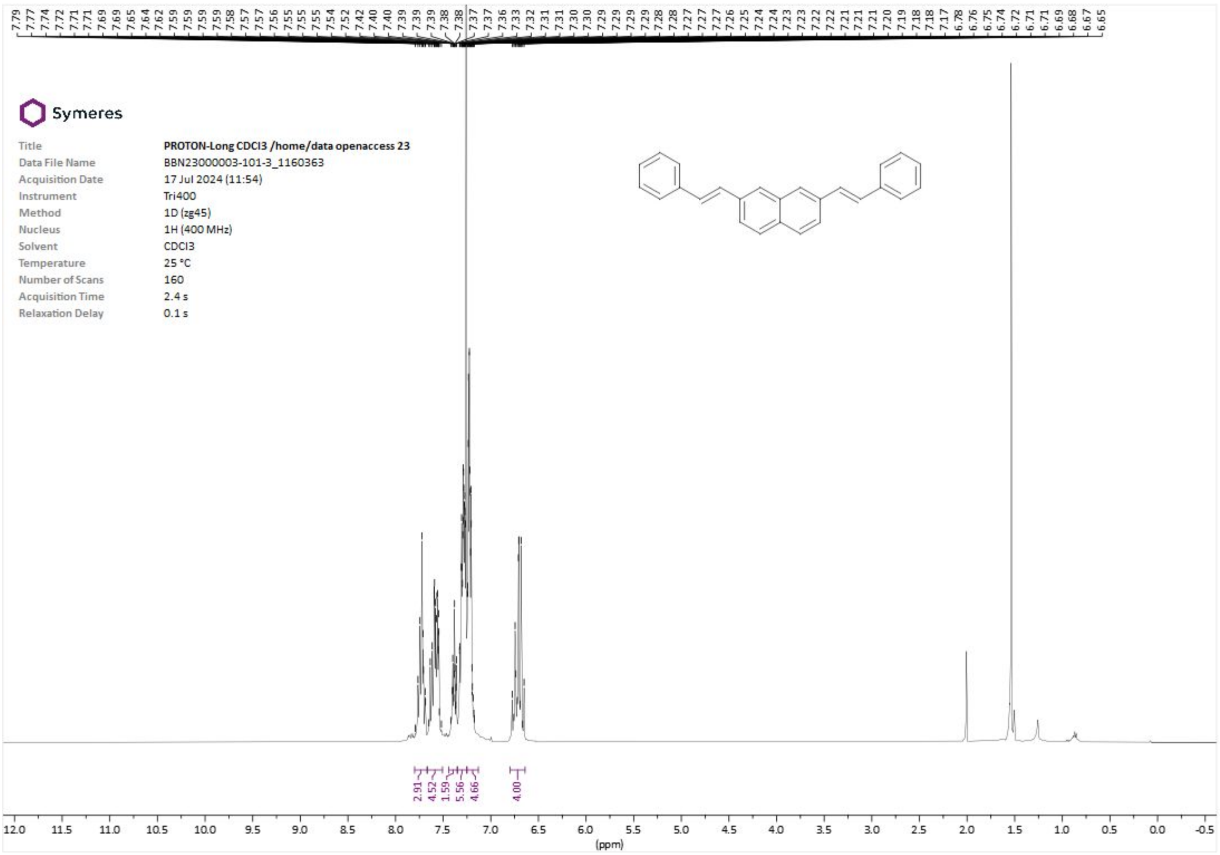

^{1}H NMR (400 MHz, $CDCl_3$) spectrum of Hexahelicene (**1**)

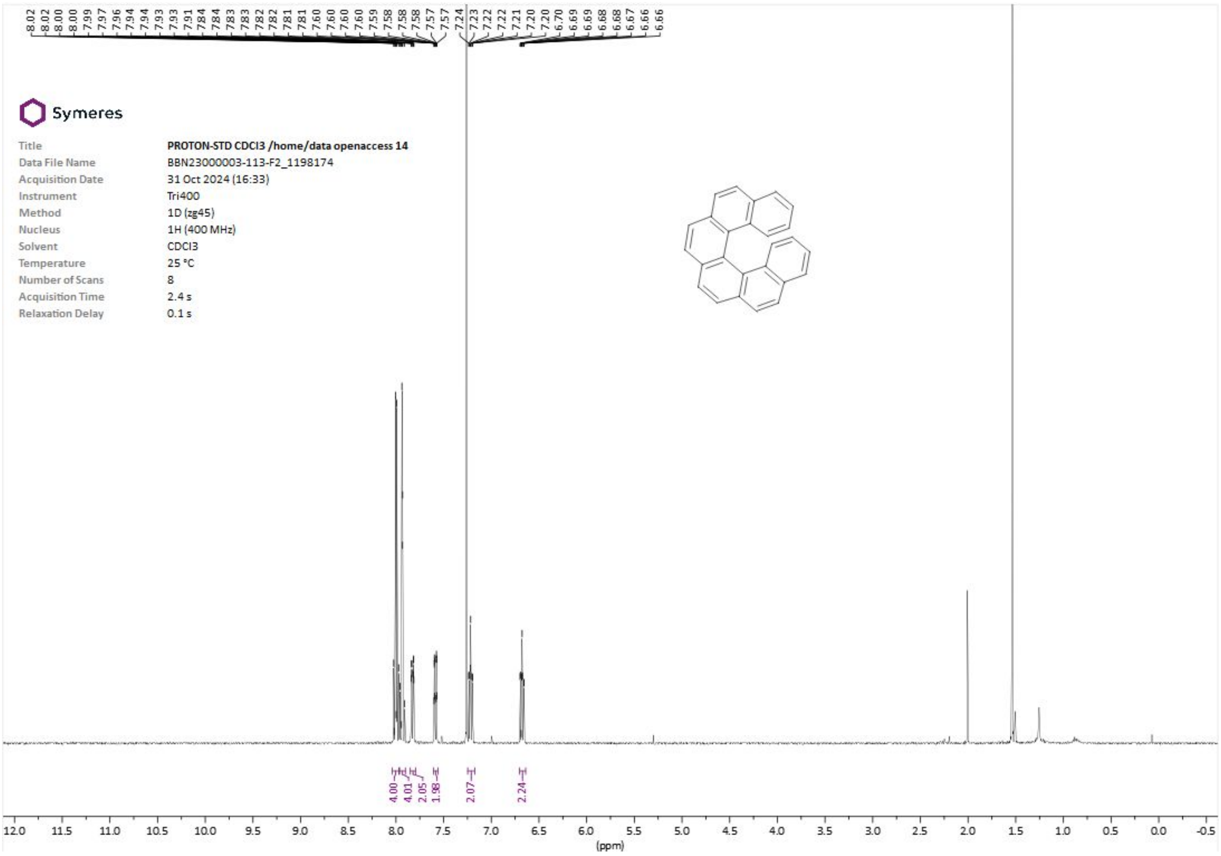

^{1}H NMR (400 MHz, $CDCl_3$) spectrum of 1-bromohexahelicene (**4**)

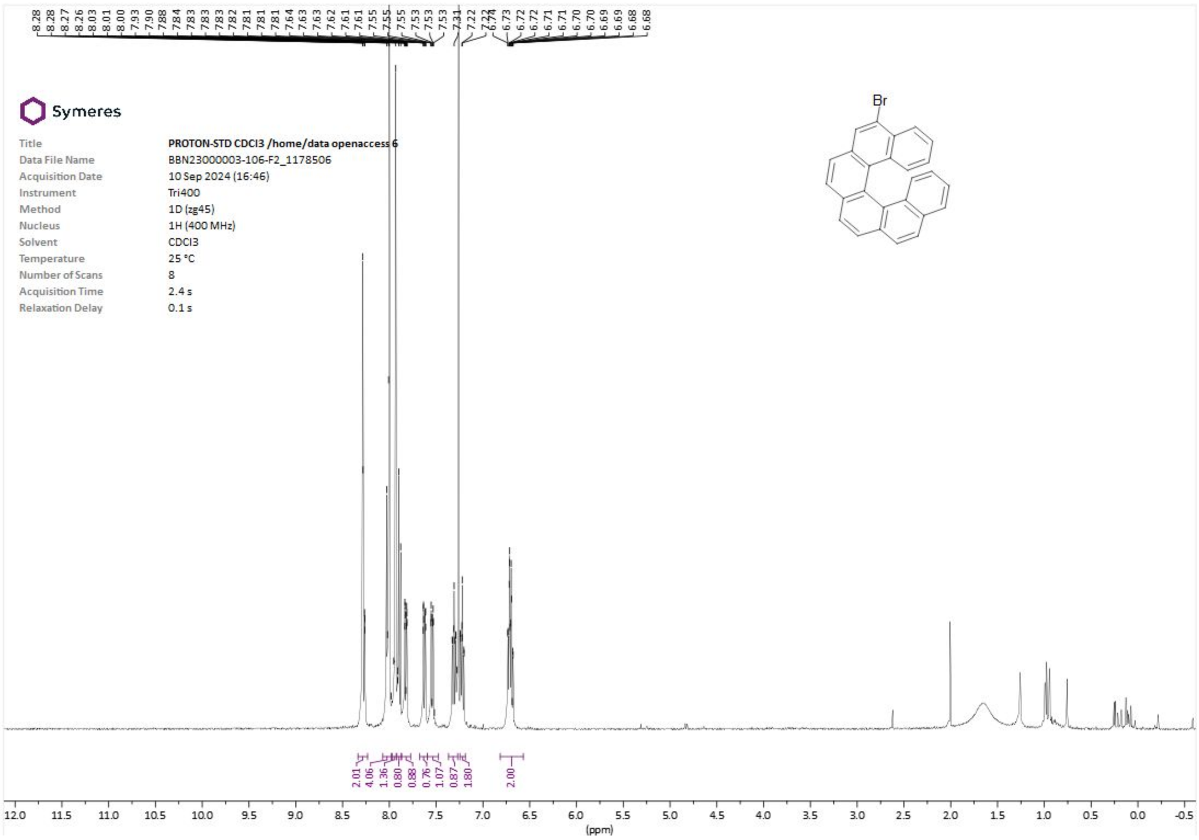

^{1}H NMR (400 MHz, $CDCl_3$) spectrum of hexahelicene-1-carbaldehyde (**5**)

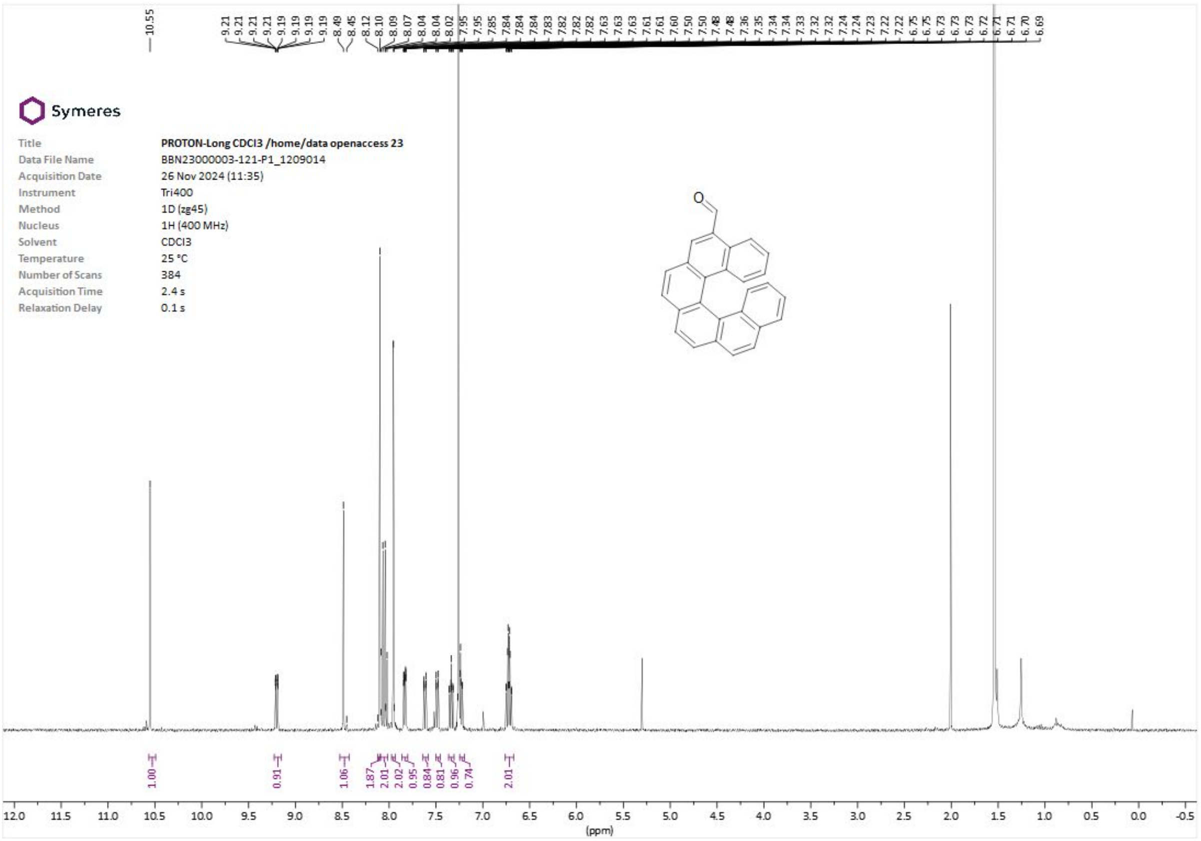

ONE-BY-ONE COMPARISON BETWEEN EXPERIMENTAL AND COMPUTATIONAL RESULTS

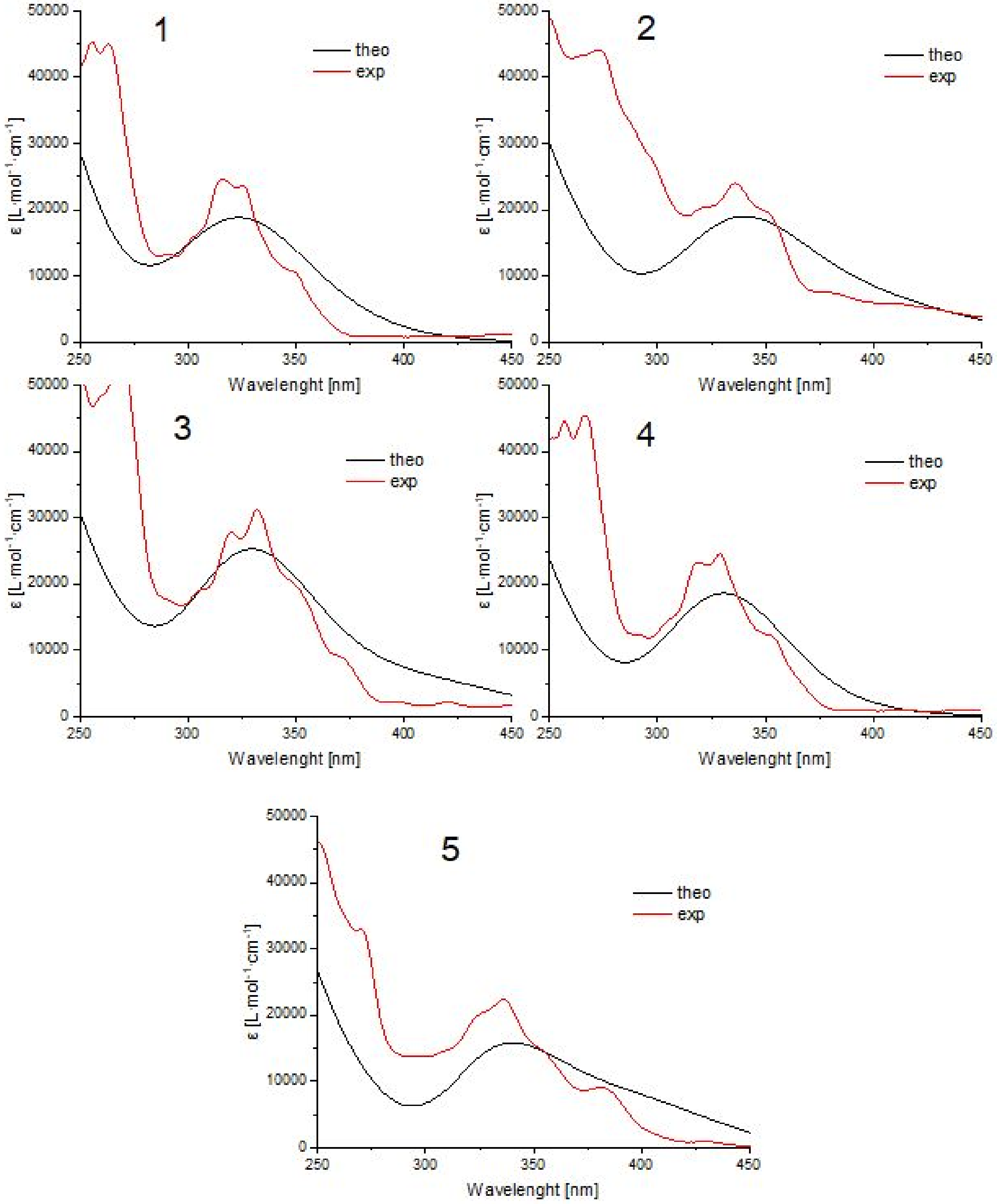

Figure S1. Comparison between experimental and computational absorption spectra. Theoretical results are redshifted by 60 nm. Numbers refers to the numbering code in Figure 1 of the main text.

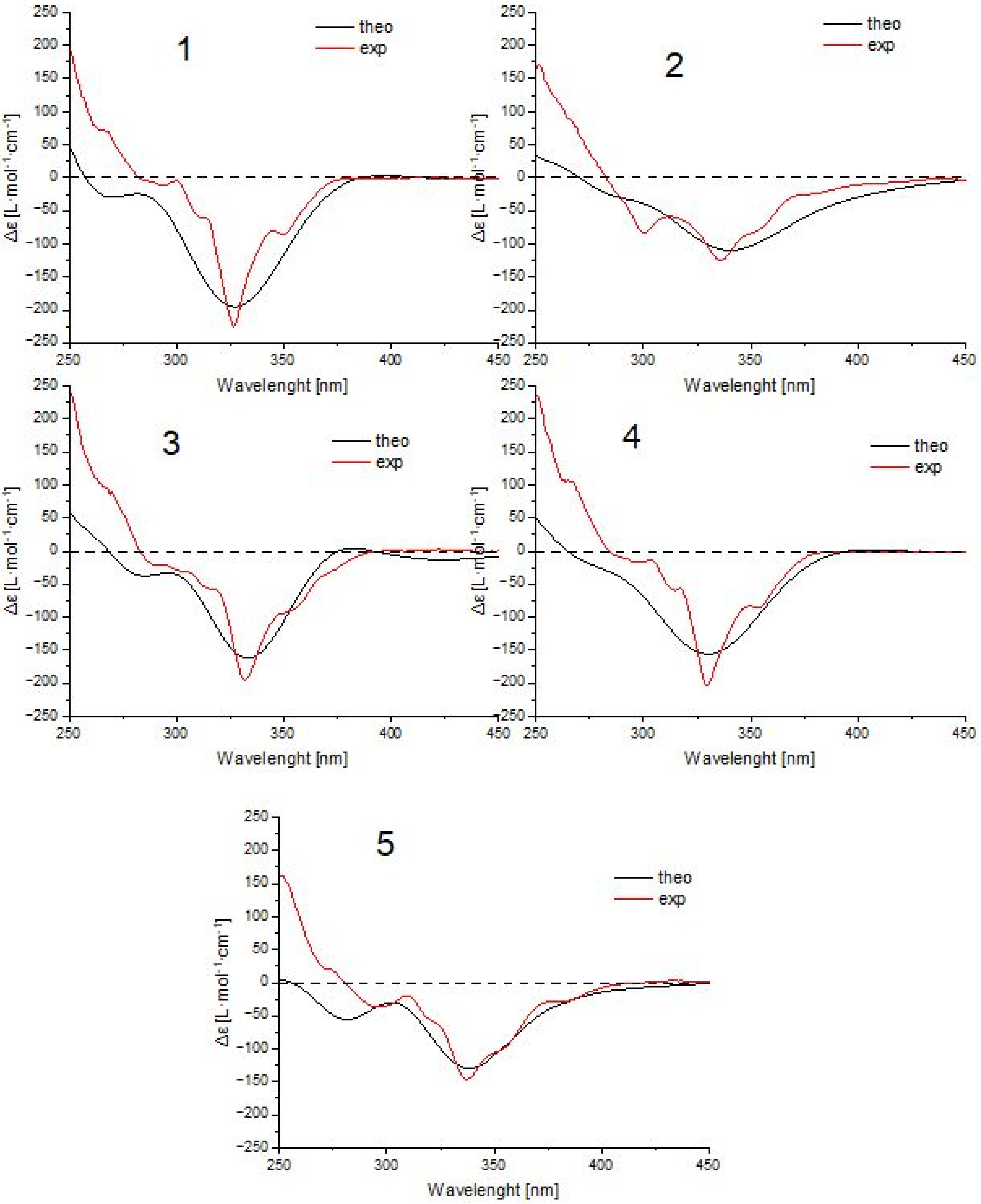

Figure S2. Comparison between experimental and computational ECD spectra. Theoretical results are redshifted by 60 nm. Numbers refers to the numbering code in Figure 1 of the main text.

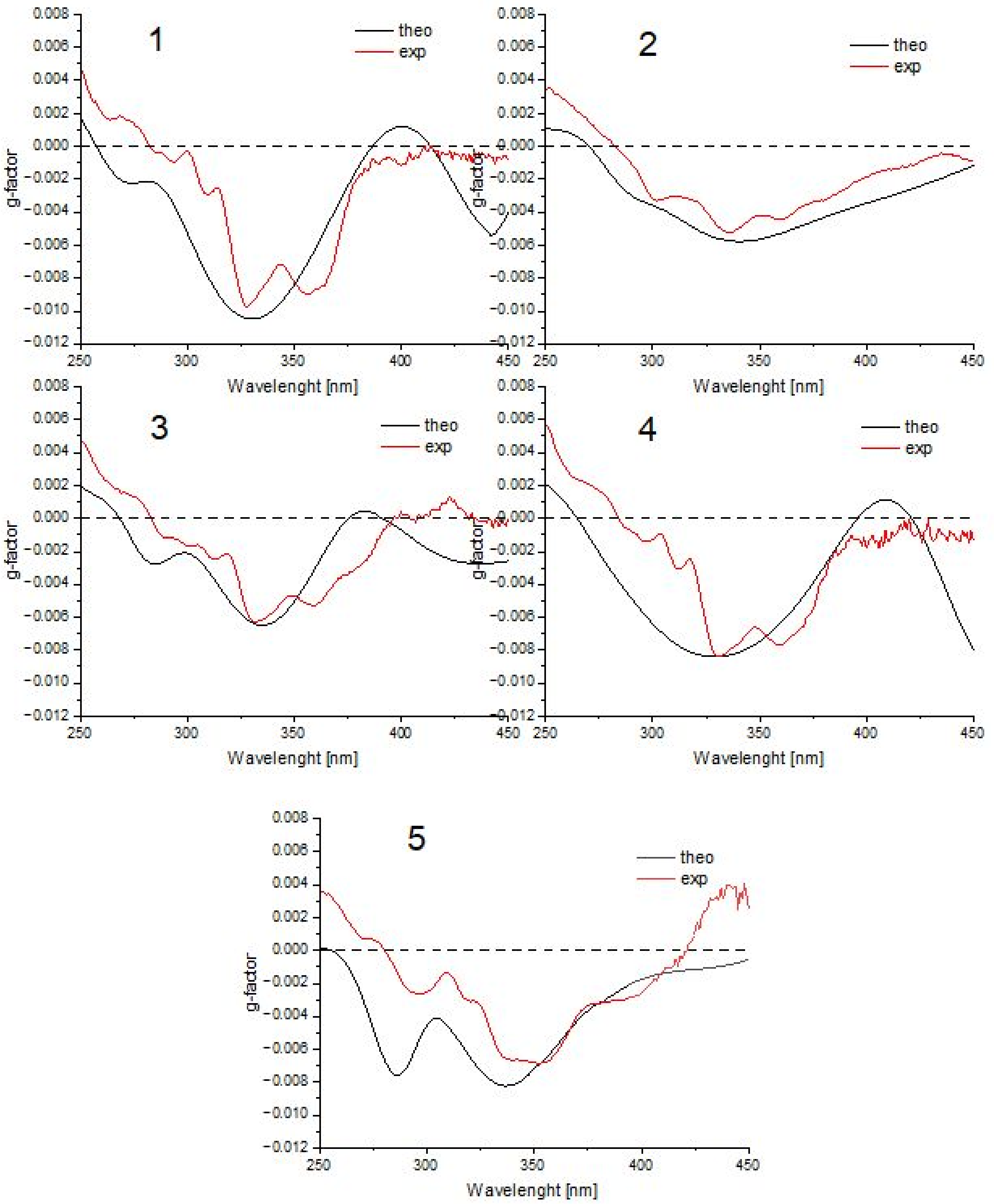

Figure S3. Comparison between experimental and computational g-factors. Theoretical results are redshifted by 60 nm. Numbers refers to the numbering code in Figure 1 of the main text.

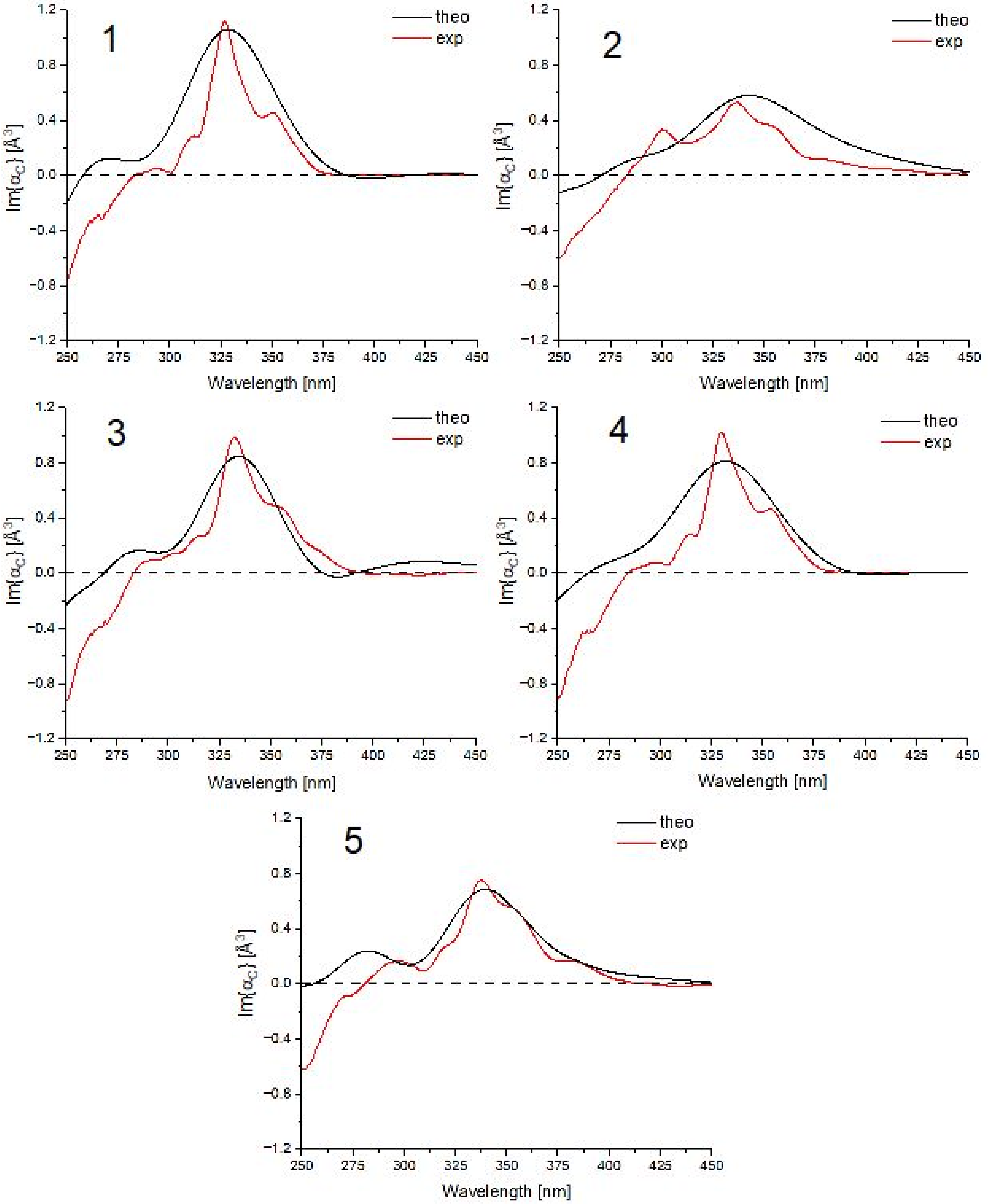

Figure S4. Comparison between experimental and computationally estimated imaginary part of chiral polarizability. Theoretical results are redshifted by 60 nm. Numbers refers to the numbering code in Figure 1 of the main text.

CORRELATION BETWEEN CCM AND HCM

Figure S5 displays a linear relationship between CCM and HCM. The five coloured data points refers to the descriptors values of the various molecules investigated.
The line fits the data well, with a high adjusted R-square value of 0.93785, indicating that these two descriptors gives the same information about the structural chirality of the tested molecules.
Key regression statistics are shown in the inset, with line intercept being 0.08 ± 0.01 and slope 0.014 ± 0.002. This suggests a positive correlation between the two descriptors.

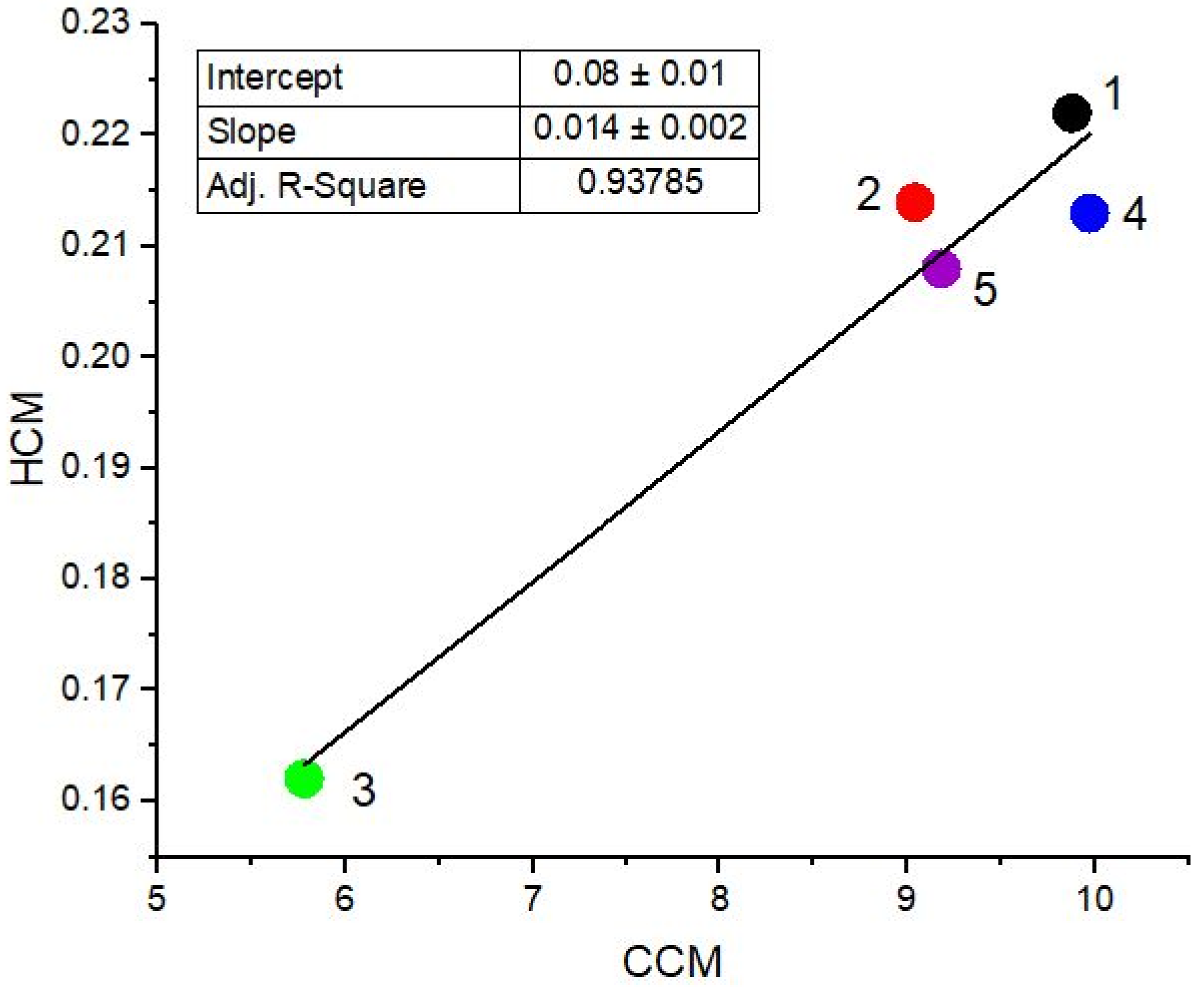

Figure S5. Correlation between CCM and HCM. Color points and numbers refer to molecules as per Figure 1 of the main text.

CONSIDERATIONS ABOUT BP86 AND CAM-B3LYP XC FUNCTIONALS

Chiroptical properties are intrinsically dependent on the nature of electronic excitations. Therefore, the selection of a computational model capable of accurately describing these transitions, including potential charge-transfer character, is critical. To validate the BP86 results presented in the main text, we performed comparative calculations using the long-range corrected CAM-B3LYP functional, which is well-established for its proficiency in modelling charge-transfer states.

CAM-B3LYP calculations have been performed using linear response TDDFT as implemented in ADF version 2025.1[1], using a TZP basis set. Excitations were evaluated to the lowest 50 states in the cluster. The UV–vis and ECD spectra convolution were obtained adopting Gaussian functions with a width at half-maximum of 10 nm. Calculations have been performed on pure helicene, amino-helicene and formyl-helicene, to test cases presenting different ground-state charge redistributions patterns. A comparison between the spectra calculated with CAM-B3LYP, the BP86 results (redshifted by 60 nm), and the experimental data is provided in Figure S6.

This analysis reveals that the CAM-B3LYP functional predicts the transition energies with significantly higher accuracy than BP86, deviating from the experimental absorption maxima by only 15–20 nm. However, this improvement in peak position is accompanied by a substantial underestimation of peak intensities. Furthermore, the corresponding g-factor is qualitatively and quantitatively incorrect compared to the experimental results. This comparison ultimately validates that the computational framework adopted in the main text (BP86) which, despite requiring a rigid shift of the spectra, provides a more balanced and faithful reproduction of the overall experimental data.

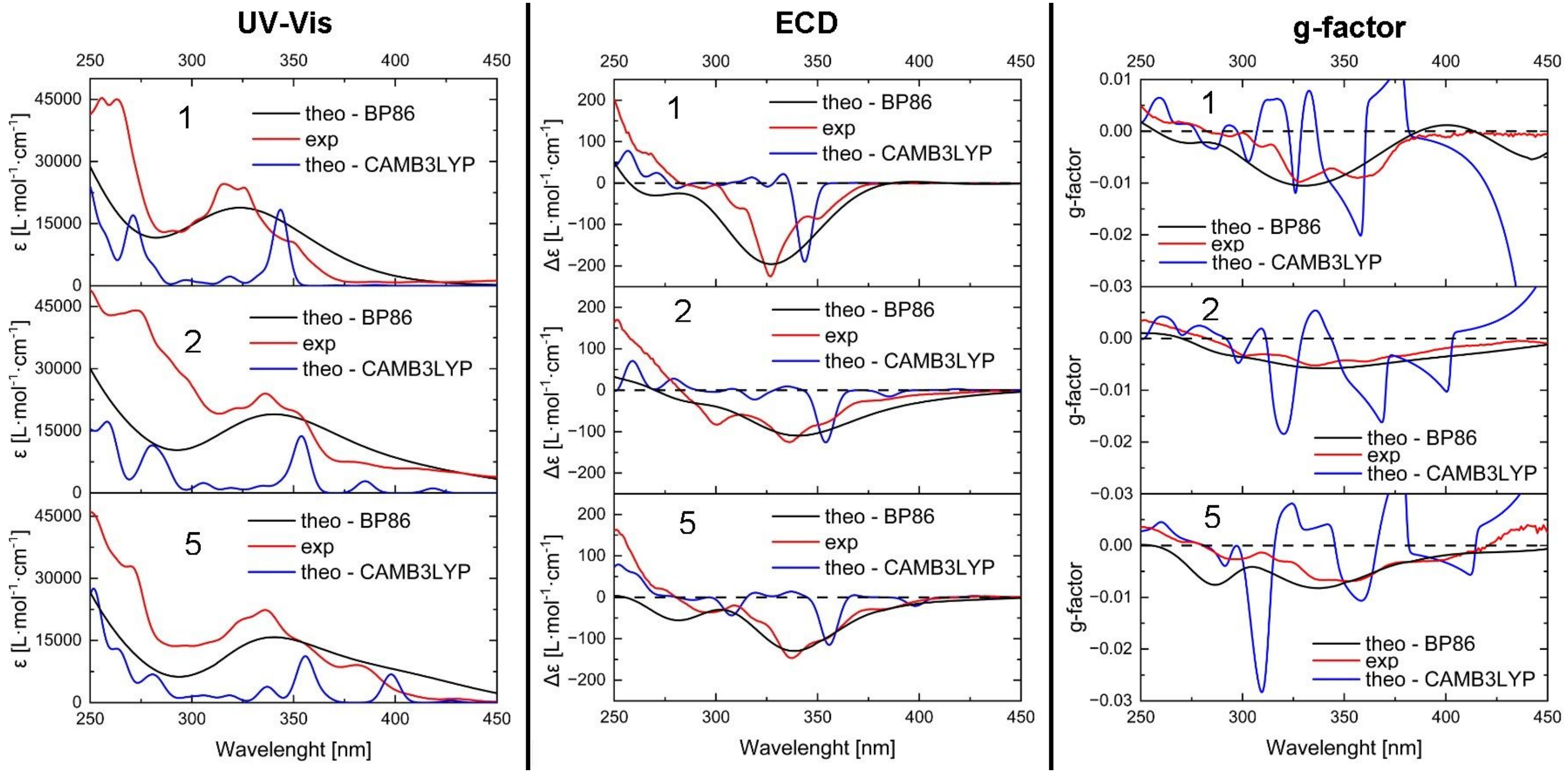

Figure S6. Comparison between experimental and computational data, obtained with two different XC functional. BP86 results are redshifted by 60 nm, CAM-B3LYP results are magnified by a factor of 10. Inset numbers refers to the numbering code in Figure 1 of the main text.

MOLECULAR ORBITAL ANALYSIS

Figure S7 shows the orbitals involved in the main low-energy transition at the CAM-B3LYP level. The single electron transition shown contributes 55%, 72%, and 75% to the total excitation for molecules 1, 2, and 5, respectively. Such transitions peak at 344 nm, 354 nm and 355 nm for molecules 1, 2, and 5, respectively. As can be seen, these excitations do not involve significant charge transfers between the substituent and the helical structure.

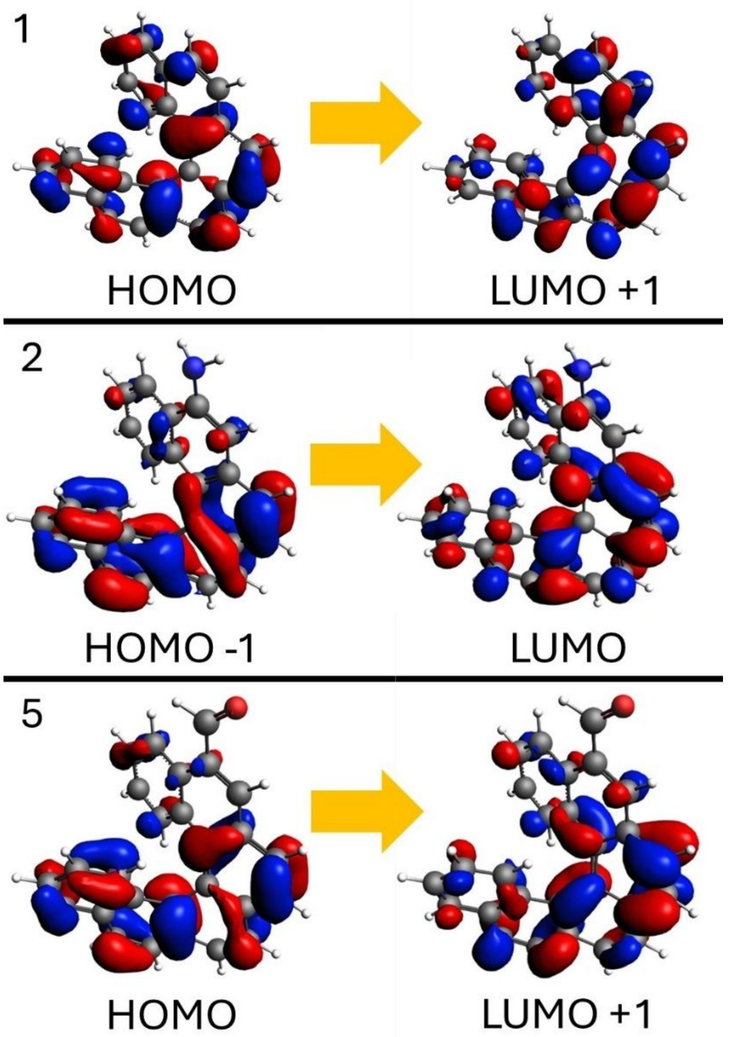

Figure S7. Graphical representation of the molecular orbitals involved in the main optical transitions. Results obtained based on CAM-B3LYP calculations. Isovalue=±0.03 a.u. Grey, white, blue and red balls represent C, H, N and O atoms respectively. Panel numbers refer to the numbering code in Figure 1 of the main text.

INDUCED CHARGE DENSITY ANALISIS

We calculate the induced charge densities as:

$$\Delta\rho(r) = \rho_{EXC}(r) - \rho_{GS}(r) \qquad \text{(S1)}$$

where $\rho_{GS}(r)$ is the ground state electron density and $\rho_{EXC}(r)$ is the electron density obtained upon optical excitation. Here the excitation comes from a laser pulse composed of three plane waves, polarized along the three main Cartesian axes, which frequencies match the molecular maximum absorption wavelength in the 300-400 nm window and field amplitudes sum up to 0.1 V/nm.

The results, presented in Figure S8, indicate that in all cases, optical excitation leads to a depletion of electron density in the carbon backbone, accompanied by an enrichment of electrons within the aromatic rings, which in turn leads to an enhancement of the ring current. However, it remains challenging to assess the specific influence of the substituents, as no systematic or discernible patterns emerge from the analysis.
It is also important to note that the charge densities were computed in real time under the influence of an external field, with the wavefront propagating along the (1 1 1) crystallographic direction. This introduces an inherent directionality to the simulation, further complicating the interpretation of substituent-dependent effects. As a future direction, we plan to investigate potential charge-transfer phenomena through a detailed analysis of the involved molecular orbitals, which may provide a clearer understanding of the substituents' roles in modulating the electronic response.

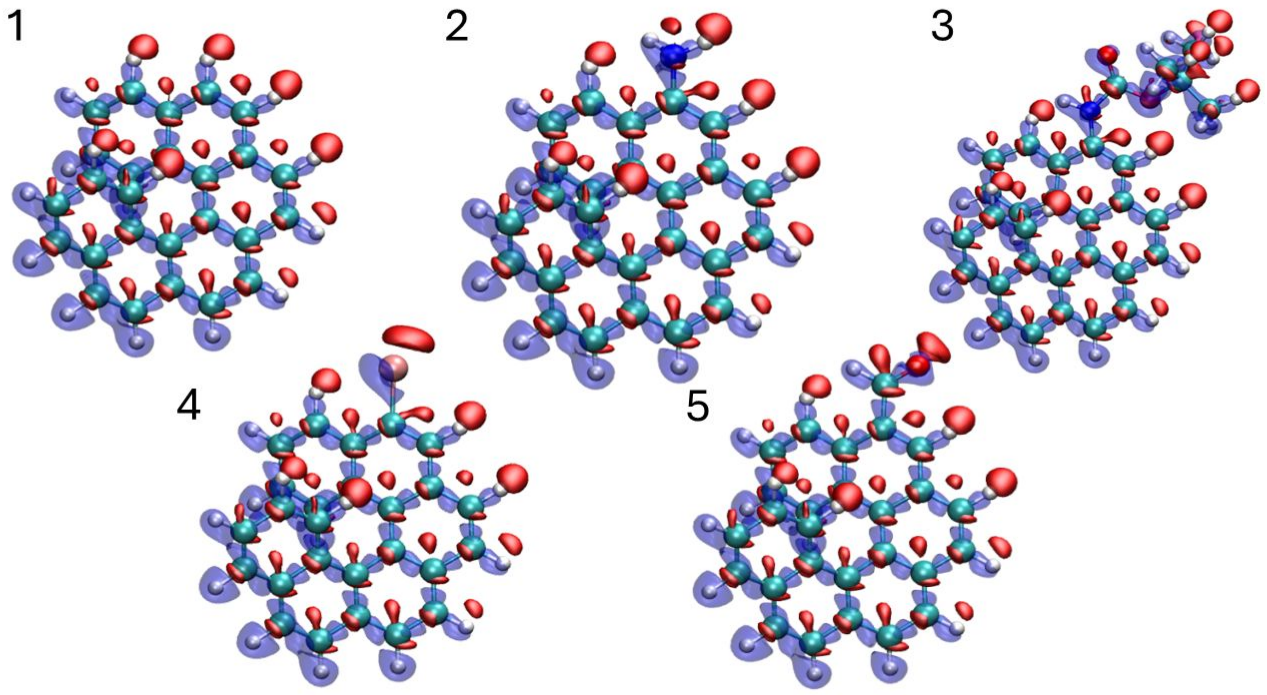

Figure S8. Induced charge densities upon optical excitation. Blue/red isosurfaces indicate areas where the depletion/excess is 0.5 e.

DFT-OPTIMIZED GEOMETRICAL STRUCTURES AND PARTIAL ATOMIC HIRSHFELD CHARGES OF THE INVESTIGATED MOLECULES

Column 1 indicates the element. Column 2-4 are the x, y and z coordinate of the atom in atomic units. Column 5 is the ground state partial atomic Hirshfeld charge,[2,3] which is defined as the atom Hirshfeld charge (calculated by the ab-initio code[4–6]) minus the number of valence electrons. Here negative values indicate a lack of electrons and thus a positive partial atomic charge. Molecular numbering defined according to Figure 1 of the main text. All geometries refer to relaxed geometrical structures as described in the Methods section.

Molecule 1

C	-1.761562	-3.179533	-0.592082	-0.002
C	-3.697252	-4.294948	-2.135533	0.003
C	0.888320	-3.652027	-1.193611	-0.004
C	-2.557934	-1.812170	1.576098	0.054
C	-3.015349	-5.988586	-4.129702	0.064
C	-6.278286	-3.818869	-1.584966	0.064
C	-0.558209	-6.726345	-4.424807	0.065
C	1.419309	-5.667107	-2.909994	0.004
C	3.897907	-6.717726	-3.047150	0.064
C	3.015359	-2.379918	0.000849	-0.007
C	5.342547	-3.764608	0.154308	0.002
C	5.741183	-5.918106	-1.416214	0.063
C	7.265176	-2.991760	1.877563	0.063
C	3.008729	0.161279	1.054940	-0.004
C	6.936423	-0.900015	3.365315	0.064
C	4.858654	0.760522	2.927676	0.004
C	4.705492	3.087350	4.302133	0.065
C	1.400935	2.221619	0.173242	-0.003
C	2.927088	4.869673	3.728060	0.064
C	1.313897	4.540359	1.583477	0.003
C	-0.269614	6.541616	0.759056	0.064
C	0.036605	2.139199	-2.136837	0.055
C	-1.440668	4.146998	-2.937536	0.061
C	-1.648199	6.352488	-1.453511	0.065
C	-5.092313	-1.433826	2.113081	0.063
C	-6.978530	-2.393981	0.491356	0.065
H	-1.146445	-1.076880	2.875353	-0.052
H	-4.505694	-6.779904	-5.313888	-0.063
H	-7.710407	-4.658059	-2.808443	-0.062
H	-0.049733	-8.161162	-5.815298	-0.062
H	4.242885	-8.268477	-4.360209	-0.064
H	7.568324	-6.868353	-1.332271	-0.063
H	8.968588	-4.142675	2.026763	-0.064
H	8.330646	-0.383319	4.792613	-0.064

H	6.068226	3.409836	5.815298	-0.062
H	2.801362	6.622400	4.805828	-0.064
H	-0.329549	8.268477	1.884399	-0.062
H	0.185828	0.476066	-3.333760	-0.053
H	-2.437875	4.019928	-4.735410	-0.066
H	-2.838651	7.913293	-2.076504	-0.065
H	-5.626522	-0.396203	3.810434	-0.067
H	-8.968588	-2.062818	0.905093	-0.065

Molecule 2

C	-1.768839	-3.311200	-0.925338	-0.002
C	-3.718180	-4.414626	-2.459944	0.004
C	0.876490	-3.802401	-1.535471	-0.002
C	-2.548994	-1.937234	1.245023	0.056
C	-3.052227	-6.116694	-4.453145	0.066
C	-6.293973	-3.918275	-1.901217	0.065
C	-0.601361	-6.874995	-4.751016	0.067
C	1.390362	-5.829391	-3.243888	0.005
C	3.861682	-6.896152	-3.381844	0.069
C	3.013970	-2.531708	-0.357669	-0.006
C	5.332503	-3.931713	-0.200036	0.007
C	5.713196	-6.095625	-1.756922	0.066
C	7.260646	-3.144888	1.512336	0.065
C	3.013751	0.004681	0.690534	0.008
C	6.945561	-1.040703	2.985079	0.068
C	4.859839	0.620772	2.557892	0.006
C	4.708254	2.936019	3.919123	0.095
C	1.408066	2.069105	-0.173942	-0.004
C	2.889515	4.734458	3.415116	-0.044
C	1.262942	4.384082	1.240395	0.010
C	-0.371786	6.332910	0.392930	0.066
C	0.085009	1.970548	-2.506943	0.052
C	-1.424801	3.939501	-3.338241	0.062
C	-1.712646	6.122805	-1.844773	0.066
C	-5.078707	-1.540686	1.791511	0.064
C	-6.977700	-2.486626	0.176442	0.067
H	-1.126707	-1.211433	2.537987	-0.053
H	-4.550530	-6.897988	-5.634175	-0.063
H	-7.737174	-4.746968	-3.119085	-0.060
H	-0.108870	-8.315745	-6.141388	-0.063
H	4.196738	-8.455069	-4.687605	-0.061
H	7.535412	-7.056218	-1.672561	-0.063
H	8.963884	-4.296093	1.667796	-0.063

H	8.354141	-0.518191	4.396574	-0.061
H	6.099489	3.278871	5.404999	-0.056
H	-0.580505	8.058789	1.498143	-0.054
H	0.296038	0.307850	-3.695091	-0.053
H	-2.387177	3.792623	-5.153216	-0.067
H	-2.941028	7.655596	-2.463187	-0.065
H	-5.598614	-0.499068	3.491084	-0.065
H	-8.963884	-2.139122	0.596131	-0.064
H	1.951360	8.455069	4.120747	-0.128
H	4.080158	7.204476	6.141388	-0.140
N	2.630357	6.864533	4.938147	0.205

Molecule 3

C	-2.116868	-4.789111	-4.801650	-0.001
C	-3.946949	-6.216940	-6.170835	0.004
C	0.555009	-5.260959	-5.249479	-0.002
C	-3.076392	-3.002994	-3.050933	0.053
C	-3.185586	-8.271881	-7.701041	0.064
C	-6.545025	-5.644306	-5.986965	0.062
C	-0.712522	-8.940286	-7.852438	0.064
C	1.185826	-7.502740	-6.613191	0.002
C	3.696669	-8.439261	-6.686871	0.067
C	2.603143	-3.744576	-4.243295	-0.008
C	4.990198	-5.018523	-3.964301	0.004
C	5.515211	-7.308687	-5.261611	0.062
C	6.894653	-4.021281	-2.363095	0.062
C	2.500315	-1.103089	-3.445771	0.003
C	6.485259	-1.827791	-1.077212	0.063
C	4.348509	-0.292279	-1.668941	0.003
C	4.204729	2.127571	-0.509690	0.083
C	0.837968	0.881536	-4.415104	-0.003
C	2.494165	3.917979	-1.282893	-0.045
C	0.944790	3.398551	-3.434860	0.007
C	-0.493696	5.375969	-4.526453	0.064
C	-0.954850	0.503317	-6.337372	0.048
C	-2.545633	2.398261	-7.156414	0.061
C	-2.282945	4.879824	-6.331047	0.062
C	-5.633064	-2.459890	-2.897630	0.061
C	-7.394739	-3.739952	-4.429399	0.067
C	3.097974	7.482549	1.843079	-0.214
C	6.146459	6.507741	5.190437	-0.097
C	7.955490	4.248022	5.344924	0.157
C	7.455576	9.086569	5.541318	0.160

C	3.918900	6.167032	6.989431	0.163
H	-1.764301	-1.985087	-1.851567	-0.051
H	-4.648718	-9.291454	-8.659615	-0.062
H	-7.829728	-6.742529	-7.135555	-0.061
H	-0.106328	-10.562632	-8.919476	-0.062
H	4.092189	-10.118088	-7.778924	-0.064
H	7.411585	-8.096829	-5.143480	-0.065
H	8.670622	-5.056031	-2.211899	-0.064
H	7.908934	-1.076572	0.203573	-0.061
H	5.586230	2.552474	0.929097	-0.041
H	-0.150167	7.342297	-4.046012	-0.060
H	-1.092132	-1.341639	-7.155573	-0.047
H	-3.973998	2.013691	-8.499111	-0.064
H	-3.414953	6.373931	-7.142986	-0.069
H	-6.272614	-0.983291	-1.617508	-0.065
H	-9.387499	-3.247615	-4.383508	-0.066
H	9.116218	4.149461	3.649377	-0.056
H	6.887417	2.474477	5.463628	-0.055
H	9.197370	4.403629	6.986986	-0.062
H	7.518847	9.565972	7.552600	-0.060
H	6.379956	10.562632	4.567182	-0.055
H	9.387499	9.099313	4.836996	-0.056
H	4.621840	5.928624	8.919476	-0.060
H	2.866614	4.464422	6.443299	-0.055
H	2.650157	7.797063	6.904645	-0.053
H	0.532243	7.229267	-0.896826	-0.128
N	2.052326	6.292393	-0.210216	0.100
O	5.179873	6.230825	2.594005	0.088
O	2.212246	9.417301	2.740304	0.276

Molecule 4

C	-1.798725	-3.057632	-0.486632	-0.002
C	-3.773719	-4.133331	-2.004696	0.003
C	0.829449	-3.585460	-1.128498	-0.004
C	-2.535547	-1.667437	1.688499	0.055
C	-3.143932	-5.850788	-3.997212	0.063
C	-6.336515	-3.587864	-1.430952	0.063
C	-0.706779	-6.649644	-4.312405	0.065
C	1.310837	-5.622393	-2.828498	0.004
C	3.778813	-6.699722	-2.981776	0.064
C	2.987424	-2.319804	0.000303	-0.007
C	5.307060	-3.714122	0.150989	0.002
C	5.657150	-5.897389	-1.388682	0.063

C	7.252632	-2.910159	1.835549	0.061
C	3.001257	0.234996	0.990557	-0.003
C	6.946551	-0.786808	3.284934	0.062
C	4.862168	0.862590	2.833035	0.003
C	4.687132	3.189533	4.188796	0.075
C	1.379560	2.268752	0.083838	-0.006
C	2.854079	4.911587	3.608952	0.005
C	1.220454	4.602127	1.468600	0.010
C	-0.419974	6.541713	0.617093	0.067
C	0.045634	2.134451	-2.239967	0.052
C	-1.483676	4.086733	-3.074435	0.059
C	-1.773715	6.287646	-1.603368	0.062
C	-5.053406	-1.221372	2.248907	0.061
C	-6.978380	-2.135796	0.645917	0.064
H	-1.093140	-0.966847	2.973549	-0.053
H	-4.661413	-6.610728	-5.167158	-0.064
H	-7.803846	-4.391592	-2.636075	-0.062
H	-0.247584	-8.100853	-5.702608	-0.064
H	4.093418	-8.269791	-4.278965	-0.065
H	7.474571	-6.867159	-1.319025	-0.065
H	8.954582	-4.061485	1.996323	-0.066
H	8.351133	-0.246586	4.692911	-0.066
H	6.031413	3.561269	5.702608	-0.064
H	-0.549961	8.269791	1.725557	-0.046
H	0.260799	0.463149	-3.414617	-0.054
H	-2.457419	3.917426	-4.881079	-0.068
H	-3.009558	7.808537	-2.236253	-0.067
H	-5.544847	-0.166279	3.948376	-0.065
H	-8.954582	-1.748108	1.076497	-0.066
Br	2.618115	7.879260	5.626528	-0.005

Molecule 5

C	-1.807530	-3.211202	-1.034524	-0.003
C	-3.769747	-4.315469	-2.550150	0.001
C	0.826068	-3.718608	-1.662708	-0.006
C	-2.559789	-1.808794	1.126334	0.054
C	-3.122980	-6.045312	-4.525753	0.062
C	-6.337833	-3.783552	-1.989359	0.062
C	-0.678575	-6.825361	-4.827297	0.065
C	1.325320	-5.764536	-3.348208	0.003
C	3.800655	-6.823004	-3.492655	0.058
C	2.973092	-2.422733	-0.538049	-0.007
C	5.308954	-3.795863	-0.390917	-0.002

C	5.674820	-5.989603	-1.911252	0.062
C	7.269229	-2.965551	1.266686	0.060
C	2.984342	0.147703	0.423780	-0.012
C	6.971466	-0.826298	2.688828	0.057
C	4.876659	0.813621	2.231997	0.002
C	4.706098	3.152079	3.538914	0.041
C	1.351578	2.173486	-0.491801	-0.003
C	2.863267	4.916457	3.013413	0.020
C	1.213718	4.525600	0.865983	-0.002
C	-0.410701	6.456703	-0.052277	0.062
C	0.016314	1.988033	-2.811705	0.052
C	-1.509816	3.923569	-3.686092	0.059
C	-1.775507	6.157250	-2.263553	0.060
C	-5.082543	-1.377728	1.675665	0.059
C	-6.995591	-2.319320	0.073882	0.062
C	2.727866	7.197837	4.643187	-0.090
H	-1.124846	-1.089209	2.409465	-0.053
H	-4.631010	-6.827381	-5.693179	-0.066
H	-7.795486	-4.606321	-3.193152	-0.063
H	-0.202256	-8.284838	-6.202962	-0.065
H	4.127245	-8.401214	-4.777327	-0.066
H	7.501716	-6.940866	-1.840397	-0.067
H	8.975678	-4.111130	1.419430	-0.067
H	8.387026	-0.258220	4.073950	-0.069
H	6.055128	3.569640	5.042405	-0.065
H	-0.536524	8.244228	0.956169	-0.056
H	0.236379	0.297430	-3.957619	-0.052
H	-2.484468	3.722456	-5.488938	-0.068
H	-2.991946	7.677318	-2.935233	-0.068
H	-5.587599	-0.314355	3.365862	-0.067
H	-8.975678	-1.942076	0.495209	-0.067
H	1.012680	8.401214	4.419841	-0.051
O	4.330161	7.782227	6.202962	0.232

REFERENCES

(1) Baerends, E. J.; Aguirre, N. F.; Austin, N. D.; Autschbach, J.; Bickelhaupt, F. M.; Bulo, R.; Cappelli, C.; van Duin, A. C. T.; Egidi, F.; Fonseca Guerra, C.; Förster, A.; Franchini, M.; Goumans, T. P. M.; Heine, T.; Hellström, M.; Jacob, C. R.; Jensen, L.; Krykunov, M.; van Lenthe, E.; Michalak, A.; Mitoraj, M. M.; Neugebauer, J.; Nicu, V. P.; Philipsen, P.; Ramanantoanina, H.; Rüger, R.; Schreckenbach, G.; Stener, M.; Swart, M.; Thijssen, J. M.; Trnka, T.; Visscher, L.; Yakovlev, A.; van Gisbergen, S. The Amsterdam Modeling Suite. *J. Chem. Phys.* **2025**, *162* (16), 162501. https://doi.org/10.1063/5.0258496.

(2) Hirshfeld, F. L. Bonded-Atom Fragments for Describing Molecular Charge Densities. *Theor. Chim. Acta* **1977**, *44* (2), 129–138. https://doi.org/10.1007/BF00549096.

(3) Davidson, E. R.; Chakravorty, S. A Test of the Hirshfeld Definition of Atomic Charges and Moments. *Theor. Chim. Acta* **1992**, *83* (5–6), 319–330. https://doi.org/10.1007/BF01113058.

(4) Marques, M. A. L.; Castro, A.; Bertsch, G. F.; Rubio, A. Octopus: A First-Principles Tool for Excited Electron–Ion Dynamics. *Comput. Phys. Commun.* **2003**, *151*, 60–78. https://doi.org/https://doi.org/10.1016/S0010-4655(02)00686-0.

(5) Andrade, X.; Strubbe, D.; De Giovannini, U.; Larsen, A. H.; Oliveira, M. J. T.; Alberdi-Rodriguez, J.; Varas, A.; Theophilou, I.; Helbig, N.; Verstraete, M. J.; Stella, L.; Nogueira, F.; Aspuru-Guzik, A.; Castro, A.; Marques, M. A. L.; Rubio, A. Real-Space Grids and the Octopus Code as Tools for the Development of New Simulation Approaches for Electronic Systems. *Phys. Chem. Chem. Phys.* **2015**, *17* (47), 31371–31396. https://doi.org/10.1039/c5cp00351b.

(6) Tancogne-Dejean, N.; Oliveira, M. J. T.; Andrade, X.; Appel, H.; Borca, C. H.; Le Breton, G.; Buchholz, F.; Castro, A.; Corni, S.; Correa, A. A.; De Giovannini, U.; Delgado, A.; Eich, F. G.; Flick, J.; Gil, G.; Gomez, A.; Helbig, N.; Hübener, H.; Jestädt, R.; Jornet-Somoza, J.; Larsen, A. H.; Lebedeva, I. V.; Lüders, M.; Marques, M. A. L.; Ohlmann, S. T.; Pipolo, S.; Rampp, M.; Rozzi, C. A.; Strubbe, D. A.; Sato, S. A.; Schäfer, C.; Theophilou, I.; Welden, A.; Rubio, A. Octopus, a Computational Framework for Exploring Light-Driven Phenomena and Quantum Dynamics in Extended and Finite Systems. *J. Chem. Phys.* **2020**, *152* (12), 1–32. https://doi.org/10.1063/1.5142502.
